# High-content screening identifies small molecules that remove nuclear foci, affect MBNL distribution and CELF1 protein levels via a PKC-independent pathway in myotonic dystrophy cell lines

**DOI:** 10.1093/hmg/ddt542

**Published:** 2013-10-30

**Authors:** Ami Ketley, Catherine Z. Chen, Xin Li, Sukrat Arya, Thelma E. Robinson, Javier Granados-Riveron, Inyang Udosen, Glenn E. Morris, Ian Holt, Denis Furling, Soraya Chaouch, Ben Haworth, Noel Southall, Paul Shinn, Wei Zheng, Christopher P. Austin, Christopher J. Hayes, J. David Brook

**Affiliations:** 1 School of Life Sciences, University of Nottingham, Queen's Medical Centre, Nottingham NG7 2UH, UK; 2 National Center for Advancing Translational Sciences, National Institutes of Health, Bethesda, MD 20892-3370, USA; 3 Wolfson Centre for Inherited Neuromuscular Disease, RJAH Orthopaedic Hospital, Oswestry SY10 7AG, UK; 4 Institute for Science and Technology in Medicine, Keele University, Staffordshire, UK; 5 UPMC Univ Paris 06, UM 76, Institut de Myologie and Inserm, U974 and CNRS, UMR7215, F-75013 Paris, France; 6 Molecular Devices, Eskdale Road, Winnersh Triangle, Wokingham, Berkshire RG41 5TS, UK; 7 School of Chemistry, University of Nottingham, University Park, Nottingham NG7 2RD, UK

## Abstract

Myotonic dystrophy (DM) is a multi-system neuromuscular disorder for which there is no treatment. We have developed a medium throughput phenotypic assay, based on the identification of nuclear foci in DM patient cell lines using *in situ* hybridization and high-content imaging to screen for potentially useful therapeutic compounds. A series of further assays based on molecular features of DM have also been employed. Two compounds that reduce and/or remove nuclear foci have been identified, Ro 31-8220 and chromomycin A3. Ro 31-8220 is a PKC inhibitor, previously shown to affect the hyperphosphorylation of CELF1 and ameliorate the cardiac phenotype in a DM1 mouse model. We show that the same compound eliminates nuclear foci, reduces MBNL1 protein in the nucleus, affects *ATP2A1* alternative splicing and reduces steady-state levels of CELF1 protein. We demonstrate that this effect is independent of PKC activity and conclude that this compound may be acting on alternative kinase targets within DM pathophysiology. Understanding the activity profile for this compound is key for the development of targeted therapeutics in the treatment of DM.

## INTRODUCTION

Myotonic dystrophy (DM) is the most common muscular dystrophy in adults (1). It consists of two different types, both of which are caused by repeat expansion mutations and both of which share a common molecular basis (2). Although there are some clinical differences, DM1 and DM2 are progressive neuromuscular disorders in which patients suffer from myotonia, muscle weakness and wasting, and often a variety of other symptoms including cardiac arrhythmias, diabetes, and cataracts (2).

Both forms of DM are dominantly inherited. DM1 is caused by a CTG repeat sequence located in the 3′ untranslated region of the *DMPK* gene. In the unaffected population, the repeat is present as 5–30 copies, whereas in DM1 patients it is present between 50 and several thousand copies (3–5). The severity of the disease shows a general correlation with repeat expansion length. DM2 is caused by a tetranucleotide repeat expansion (CCTG) sequence present in the first intron of another gene, *CNBP*. The repeat is present as tens of copies in the normal population and as several thousand copies in DM2 patients (6).

The molecular events underlying DM pathophysiology can be broken down into a series of stages. In both forms of DM, expansion of the repeat sequences, when transcribed, produces RNAs that aggregate and remain within the nuclei of DM patient cells where they appear as distinct foci (7–9). Two groups of proteins are affected by the CUG or CCUG repeat-containing RNAs. The Muscleblind-like proteins (MBNL) are sequestered by repeat expansion transcripts (10–12), whereas CELF1 is not sequestered, but activated in DM1 by the mutant repeat expansion RNA (13). CELF1 and MBNL proteins are involved in the regulation of alternative splicing. Transcripts, such as cardiac troponin T (*TNNT2*) (14), muscle-specific chloride channel (*CLCN1*) (15), insulin receptor (*INSR*) (16), sarcoplasmic/endoplasmic reticulum Ca^2+^-ATPase (*ATP2A1*) 1 (17) and several others (18), are mis-spliced, resulting in an imbalance of isoforms which contributes to the DM phenotype. Multiple mechanisms are affected in response to RNA toxicity in DM cells and recent work points to a role for translational inhibition due to CUG repeat RNA-induced stress in DM1 (19,20). Evidence demonstrating alterations to chromatin structure, bi-directional transcription and non-ATG-initiated translation suggests that disruption of multiple molecular pathways may contribute to the DM phenotype (21,22).

There is currently no treatment for either form of DM, but an emerging understanding of the molecular basis of these disorders allows the rational development of assays to identify therapeutic compounds not currently available for the treatment of DM. This has led to the publication of biochemical and oligonucleotide-based approaches to identify compounds that target the repeat expansion sequences and disrupt the interaction between MBNL and the repeats (23–28). Other studies report the use of cell-based assays in conjunction with fluorescent splicing reporters for compound screens (29,30). In the present study, we describe a medium throughput screen for small molecules that may have therapeutic benefit in DM. We have established a compound screening protocol with high-content imaging of nuclear foci in immortalized DM cells using an *in situ* hybridization-based approach. We have screened the NIH Chemical Genomics Center (NCGC) Pharmaceutical Collection (NPC), a Chembridge Diverset™ library and two smaller collections of phosphatase and kinase inhibitors to identify two compounds that eliminate nuclear foci and may have potential for further development as DM therapies.

## RESULTS

### Primary screen for compounds that remove nuclear foci

The general screening strategy we have adopted consists of four different levels to provide insight to various aspects of DM pathophysiology (Supplementary Material, Fig. S1). For the primary screen, we used an *in situ* hybridization-based protocol to identify nuclear foci in telomerized DM patient fibroblast cell lines (Supplementary Material, Fig. S2). Fibroblast cultures established from DM patients and controls were infected with an engineered lentivirus to express constitutively sufficient levels of telomerase for continuous growth in culture over extended periods of time as described previously for other cells (31). Some of the cell lines were also transduced with a lentivirus containing a construct that allows inducible expression of the myogenic regulatory factor MyoD, which when expressed converts fibroblasts into myogenic cells (31). The *in situ* hybridization screening protocol was implemented to score nuclear foci on a Molecular Devices Micro plate reader using a modified version of the granularity analysis journal. Typically fibroblast cells from DM1 patients (KBTeloMyoD) have an average of three to five foci per cell, whereas fibroblast cells from DM2 (KagoTelo) patients have between 5 and 11 foci per cell. Data were transferred to an excel spreadsheet and for each plate in the primary screen we calculated the mean number of foci per well in the control (DMSO treatment-only) wells and scored as positive any compound-treated wells that produced a change in foci number of >2 SD from the mean.

Four different libraries were screened including 13 200 compounds from a Chembridge Diverset™ library, 2724 compounds from the NPC library and two smaller libraries from Enzo Life Sciences consisting of 80 kinase inhibitors and 33 phosphatase inhibitors. Supplementary Material, Table S1 shows the numbers of compounds screened and the numbers taken forward to secondary and further assays. Following the primary screen 364 small molecules were chosen for secondary validation from the Chembridge Diverset™ library and 120 from the NPC library. Three compounds were selected for further analysis from the kinase inhibitor library. None of the compounds in the phosphatase inhibitor library produced a significant change in foci.

### Secondary screens for compounds to remove or reduce nuclear foci

Compounds that changed nuclear foci number by >2 SD from the mean of the DMSO-treated control wells were cherry-picked for a confirmation study. These compounds were screened in a 12-point titration ranging from 200 pm to 40 μm in duplicates to determine their concentration response on reduction or increase of foci. Following *in situ* hybridization, images were collected on the Molecular Devices Micro plate reader and analysed to score nuclear foci number. All images from the secondary screen were also confirmed by direct visualization by more than one individual. In addition, compounds were tested for any effect on cytotoxicity, cell viability and apoptosis using the Promega ApoTox-Glo Triplex assay to determine whether the reduction in nuclear foci was due to general compound toxicity to the cells.

Of the 120 compounds from the NPC library, 17 produced a significant increase in foci in either DM1 or DM2 cells, or both. These included meclocycline which appeared specific for DM1, teroxironum and topotecan, both of which had a more marked effect on DM2 foci. Merbromin produced a significant increase in foci, for both DM1 and DM2 (Supplementary Material, Fig. S3). From this library gemcitabine (a nucleoside analogue) and chromomycin A3 (a glycosidic antibiotic) reduced foci in both DM1 and DM2 cells. Multiple other nucleoside analogues tested had no effect (Supplementary Material, Fig. S4). Three of the 80 compounds from the kinase inhibitor library; GF 109203X, hypericin and Ro 31-8220 produced a reduction in foci in the primary screen. Of these, GF 109203X only produced a reduction in foci numbers at the very highest concentration. Most of the compounds were excluded from further consideration, including the 364 hits from the Chembridge library, because either they failed to replicate in the secondary screen or they affected cell viability, produced cytotoxicity or apoptosis over the efficacious concentration range.

**Figure 1.**
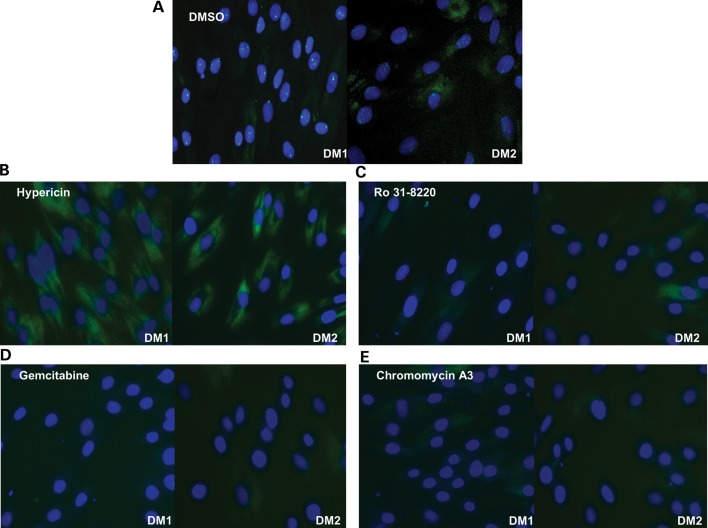
Compound treatments reduce nuclear foci. (**A–E**) Images of KBTeloMyoD fibroblast cells (DM1) and KagoTelo fibroblast cells (DM2) following RNA FISH using Cy3-(CAG)10 probe (green) for the localization of the mutant transcript and Hoechst (blue) to indicate the cell nucleus. Treatment with (A) DMSO, (B) hypericin (4.4 μm), (C) Ro 31-8220 (4.4 μm), (D) gemcitabine (4.4 μm) and (E) chromomycin A3 (4.4 μm).

**Figure 2.**
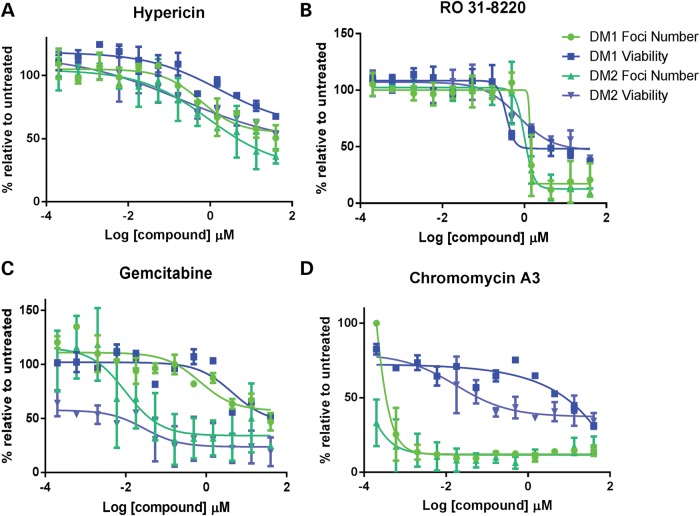
Compound treatment and toxicity profiles. Standard concentration curves showing the effect of compound treatments in KBTeloMyoD cells (DM1) and KagoTelo cells (DM2) in which the percentages of foci (green) and cell viability (blue) are represented relative to DMSO treatment on the ‘*y*’-axis. Compound concentration (log μm) is indicated on the ‘*x*’-axis for hypericin (**A**), Ro 31-8220 (**B**), gemcitabine (**C**) and chromomycin A3 (**D**).

Following the secondary screening four compounds demonstrated a significant reduction in foci number over the dilution range (Fig. 1A–E). Two of the compounds identified; gemcitabine and hypericin showed toxicity profiles that matched the disappearance of foci. Ro 31-8220 and chromomycin A3 reduced foci numbers at concentrations that did not have a fully matching toxic effect although the viability of the cells was reduced relative to controls (Fig. 2A–D). Chromomycin A3 showed the clearest difference between reduction in foci and toxicity over 2 day exposure (Figs 1E and 2D), whereas Ro 31-8220 showed a higher toxicity overall but a sharper reduction in foci over a narrow concentration range (Figs 1C and 2B). To assess the effects on other DM cell lines compounds were applied at three concentrations to three different DM1-derived myoblast lines; DM15, DM16 and DM1400 (25) and similar results were obtained (Supplementary Material, Fig. S5). Thus, Ro 31-8220 and chromomycin A3 were chosen for further analysis. To assess whether these compounds affected other aspects of DM pathophysiology they were subjected to four additional assays to examine nucleo-cytoplasmic MBNL protein distribution, alternative splicing, mutant transcript location and levels, and *in vivo* effects on a zebrafish CUG expansion model.

### Effect of compounds on MBNL1 nucleo-cytoplasmic distribution

**Figure 3.**
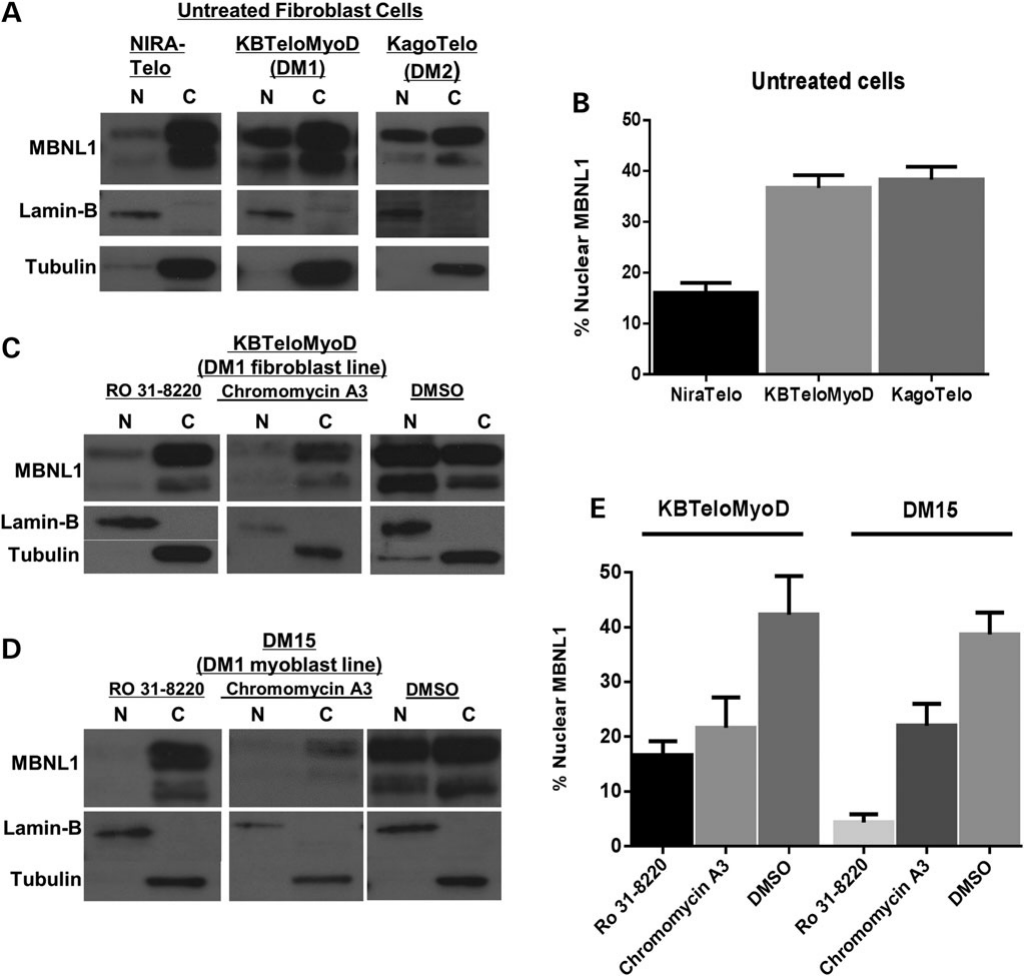
The subcellular distribution of MBNL1. (**A**) Western blots showing the distribution of MBNL1 in nuclear (N) and cytoplasmic (C) compartments of cell lines NIRATelo (control), KBTeloMyoD (DM1) and KAGOTelo (DM2). (**B**) Histograms show data compiled from intensity scans of triplicate blots for each cell line, normalized against values for lamin-B and tubulin. (**C** and **D**) Western blots showing the distribution of MBNL1 in nuclear (N) and cytoplasmic (C) compartments of fibroblast cell line KBTeloMyoD (DM1) (C) and DM1 myoblast cell line DM15 (D) following treatment with Ro 31-8220 (10 μm), chromomycin A3 (40 nm) and DMSO. (**E**) Histograms are shown of data compiled from intensity scans of triplicate blots for cell lines KBTeloMyoD and DM15 following treatment with Ro 31-8220 (10 μm), chromomycin A3 (40 nm) and DMSO normalized against values for lamin-B and tubulin.

The consensus model of DM pathogenesis suggests that MBNL proteins are sequestered in nuclear foci and as a consequence alternative splicing is impaired (32). Thus, we used western blots to examine the distribution of MBNL1 protein in nuclear and cytoplasmic fractions of DM cells before and after treatment with chromomycin A3 and Ro 31-8220. Initial analysis revealed that DM cells show a greater proportion of MBNL1 in the nuclear fraction compared with non-DM cells. In both DM1 and DM2 cells the proportion of nuclear MBNL1 was at least 50% greater than observed in non-DM cells (Fig. 3A and B). Treatment with chromomycin A3 and Ro 31-8220 resulted in reduced total protein levels in KBTeloMyoD (DM1 fibroblast) and DM15 (DM1 myoblast) cell extracts (Supplementary Material, Table S2). Treatment of DM1 fibroblasts (Fig. 3C) and myoblast cells (Fig. 3D) with each compound significantly altered the ratio of nuclear to cytoplasmic MBNL1 compared with that observed in DMSO-treated DM1 cells (Fig. 3E) with the ratios being more similar to those observed in non-DM cells. To examine the effect of compound treatment on the MBNL1 protein content of nuclear foci, we performed immunocytochemistry of DM1 cells before and after exposure to chromomycin A3 and Ro 31-8220. Consistent with their effect on RNA foci and the depletion of MBNL1 from the nuclear compartment of DM cells, both compounds significantly reduced MBNL1 in foci (Supplementary Material, Fig. S6). Thus, nuclear foci disappear as MBNL1 is depleted from the nucleus of DM cells.

### Effect of compounds on alternative splicing

**Figure 4.**
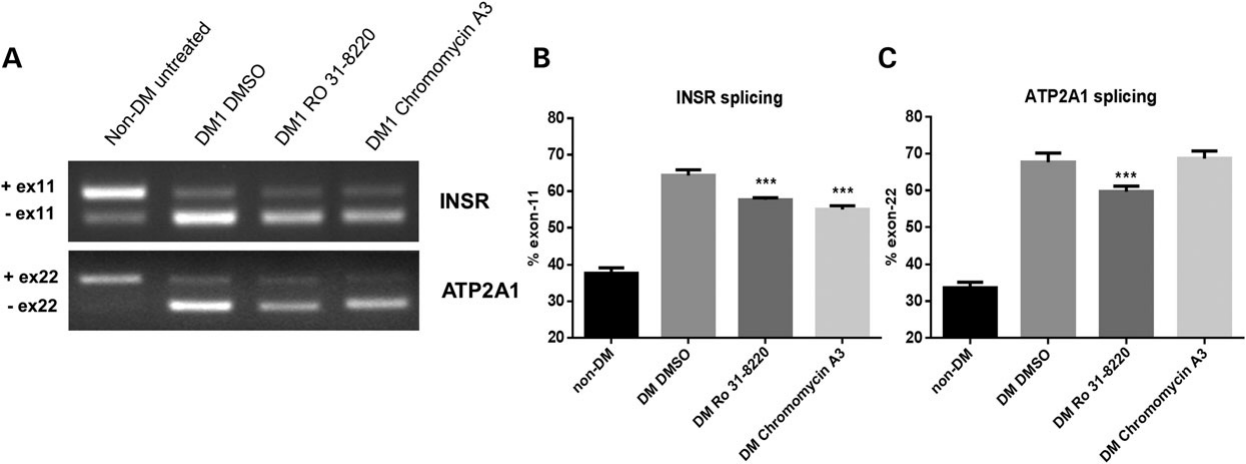
The effect of compound treatment on INSR and ATP2A1 alternative splicing. (**A**) Ethidium-stained agarose gels show RT–PCR fragment analysis of INSR and ATP2A1 splicing in DM differentiated fibroblast cell lines compared with non-DM differentiated fibroblast cells following treatment with Ro 31-8220 (10 μm), chromomycin A3 (40 nm) and DMSO (control). (**B**) Histograms show the percentage of INSR transcripts with the minus exon 11 isoform following compound treatment. (**C**) Histograms show the percentage of ATP2A1 transcripts with the minus exon 22 isoform following compound treatment. In each case, the data represent separate measurements produced for three biological replicates. Statistical analysis demonstrates the significance of compound treatments on both INSR and ATP2A1 splicing profiles towards that of the non-DM form.

One of the best described molecular features of DM is an imbalance of alternatively spliced isoforms for various transcripts (32) and we would predict that reductions in nuclear foci and the concomitant reduction in nuclear sequestration of MBNL would reverse splicing abnormalities observed in DM. Thus, we have selected two examples of MBNL-dependent splicing abnormalities to test the effect of compound treatment. INSR mis-splicing has been linked to the insulin resistance phenotype in DM patients (33). MBNL1 is the primary determinant in INSR splicing and although CELF1 does contribute, it is thought to be secondary to the role of MBNL1 (34). Work from others has shown that one of the most notable splicing changes in DM1 is the imbalance of *ATP2A1* transcript isoforms (17,18), which is MBNL1 dependent in muscle cells (35). We used assays for both of these transcripts as a read-out of the effect of drug treatment to determine whether chromomycin A3 and Ro 31-8220 reverse DM-associated alternative splicing defects. DM1 fibroblast cells with inducible MyoD were differentiated to myoblasts and treated with chromomycin A3 and Ro-31-8220 at concentrations of 40 nm and 10 μm respectively, for 48 h, and the resulting splicing profiles analysed. In DM1 cells *INSR* is mis-spliced such that there is an increase in the minus exon 11 isoform (33). Following compound treatment with both chromomycin A3 and Ro 31-8220 the minus exon 11 isoform was significantly reduced when compared with DMSO-only treated DM1 samples (Fig. 4A and B). *ATP2A1* is mis-spliced in DM1 differentiated fibroblasts with a substantial over-representation of the exon 22-minus isoform (18), whereas the *ATP2A1* exon 22-plus transcript predominates in non-DM differentiated cells (Fig. 4A and C). Chromomycin A3 treatment did not produce a significant reduction in the minus exon 22 form of *ATP2A1*. However, Ro 31-8220 produced a significant decrease (*P*–value <0.0002) in the relative proportion of the exon 22-minus *ATP2A1* transcripts in the DM cell line (Fig. 4A and C).

The data suggest that treatment with both compounds can affect DM associated mis-splicing in *INSR*, although only Ro 31-8220 significantly shifts *ATP2A1* splicing towards the distribution of non-DM cells.

### Effect of compounds on the mutant transcript

**Figure 5.**
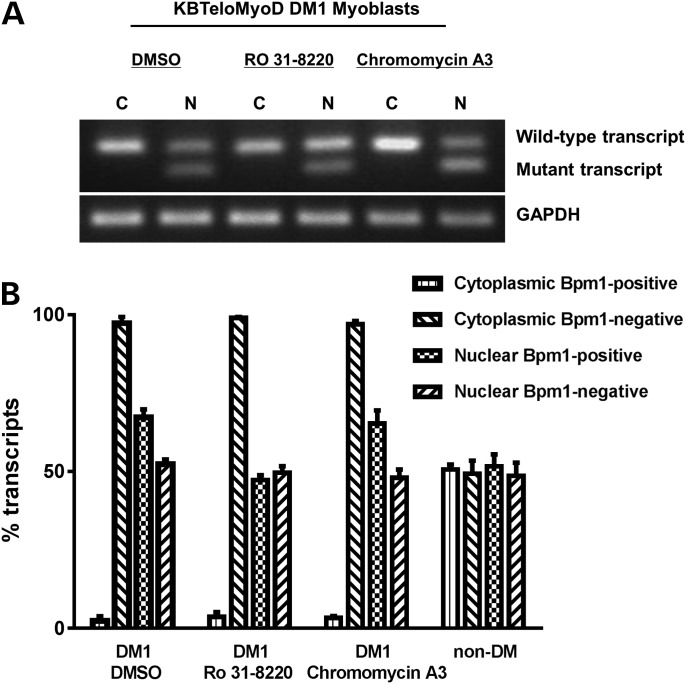
The effect of compound treatment on repeat expansion transcripts. The relative proportions of the mutant and wild-type DMPK transcripts in KBTeloMyoD differentiated fibroblast cells (DM1) were assessed following treatments with Ro 31-8220 (10 μm), chromomycin A3 (40 nm) and DMSO control. (**A**) Ethidium bromide-stained gel showing RT–PCR products from nuclear (N) and cytoplasmic (C) RNA fractions of KBTeloMyoD cells following the amplification and *BpmI* restriction enzyme digestion of a fragment of DMPK. GAPDH is used as a loading control. (**B**) Histograms showing the relative proportions of mutant (*BpmI* positive) and wild-type (*BpmI* negative) nuclear DMPK transcripts expressed as a percentage of the total nuclear DMPK transcripts. Quantitative RT–PCR was conducted using Genescan analysis of areas under the peaks following amplification of DMPK and *BpmI* restriction enzyme digestion.

To determine whether Ro 31-8220 and chromomycin A3 affect the mutant repeat expansion transcripts, we utilized an R/T PCR assay developed previously (7) which distinguishes the mutant and wild-type DMPK transcripts based on the presence or absence of a coding *BpmI* polymorphism in conjunction with nuclear and cytoplasmic RNA fractionation. Initially, products were visualized on ethidium-stained gels (Fig. 5A) to compare mutant and wild-type DMPK transcripts. Analysis of RNA from DM1 cell line KBTeloMyoD showed that although the mutant transcript was present in the nuclear fractions there was no indication of the mutant transcript in the cytoplasmic fractions before and after treatment with chromomycin A3 and Ro 31-8220 (Fig. 5A). To provide quantitative confirmation of this result and measure the relative proportion of mutant and wild-type DMPK transcripts in the nucleus and cytoplasm, quantitative RT–PCR was performed using Genescan analysis following amplification and *BpmI* restriction enzyme digestion (Fig. 5B). Around 10% of the population are informative for the *BpmI* polymorphism embedded within *DMPK* and cell line SBTeloMyoD was established from a person who did not have DM but who is heterozygous for the *BpmI* polymorphism. As such they provide a control for the distribution of transcripts with and without the *BpmI* polymorphism. Figure 5B shows clearly that both types of transcript, with and without the *BpmI* site, are present in nuclear and cytoplasmic compartments of non-DM cell line SBTeloMyoD. The histograms show that chromomycin A3 has no effect on the nuclear retention of the repeat expansion transcript or on the relative proportions of mutant and wild-type DMPK transcripts in the nucleus (Fig. 5B). Similarly, Ro 31-8220 does not affect the absence of the mutant transcript from the cytoplasm. However, it does produce a slight reduction in the relative amount of the mutant transcript in the nucleus of KBTeloMyoD (Fig. 5B). Although foci disappear there is no evidence from the analysis of cytoplasmic RNA to support the liberation of the mutant transcripts from the nucleus to the cytoplasm following treatment with either compound.

### The effect of compounds in a CUG-repeat expansion zebrafish model

To support the screen for DM therapeutic compounds, we have developed a transgenic CUG-repeat expansion zebrafish model, in which (CUG)_140_ repeat transcripts are injected into zebrafish embryos (Supplementary Material, Fig. S7). This model has not been characterized beyond the external phenotype; however, a recent publication describing a transient zebrafish embryo model with 91 CUG repeats demonstrates the presence of nuclear foci and a global down-regulation of muscle-specific developmental transcripts following injection of repeat RNA (36). The phenotype replicates the model used in this study suggesting its suitability as a basic *in vivo* model for DM1. Embryos were scored according to the number of somites observed beyond the yolk cell extension and the length to width ratio of the tail beyond the yolk cell extension**.** Uninjected control embryos were treated with Ro 31-8220 at 5 μm for 24 h and evaluated in the same way. There is a very mild negative effect on the phenotype of these embryos with the average somite number being 10% lower than untreated embryos. The length:width ratio effect was mild and reduced by 4% compared with untreated embryos. The effects of compound treatment *in vivo* were measured against repeat RNA-injected, non-compound-treated embryos. Because of its size chromomycin A3 did not penetrate effectively the zebrafish chorion, so it was not studied further in this assay. The CUG_140_ RNA reduced the somite number and length to width ratio by 40% compared with uninjected controls. Treatment with 5 μm Ro 31-8220 had a beneficial effect, with partial rescue of the mutant phenotype (Supplementary Material, Fig. S7E and F). Seventy embryos were scored in each category and the difference between CUG_140_ embryos and Ro 31-8220-treated zebrafish is significant for both somite number and length to width ratio with a *P*-value of <0.0001.

### The effect of compounds on foci is not mediated by PKC

The phenotypic effect of Ro 31-8220 on a zebrafish embryo model is consistent with the beneficial effect of this inhibitor on the cardiac phenotype observed in a DM1 mouse model (37). Ro 31-8220 is a PKC inhibitor and recent work indicates that inappropriate activation of the PKC pathway is a factor in DM pathogenesis (13,37).

**Figure 6.**
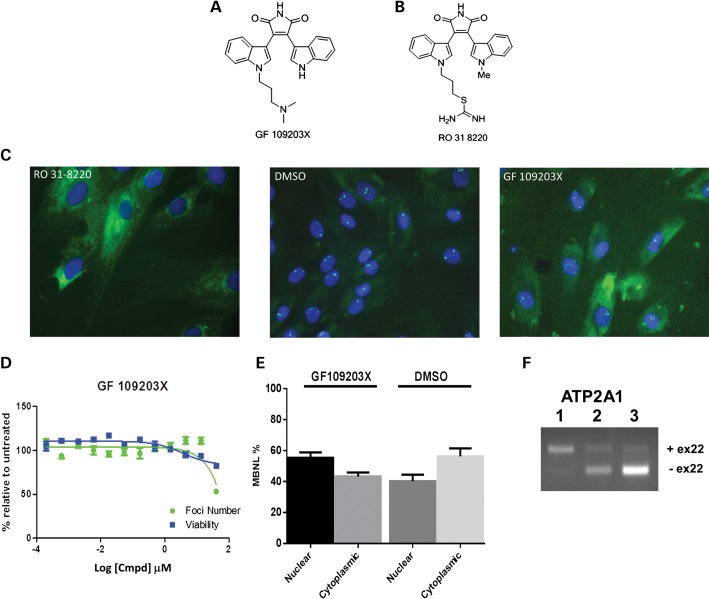
Comparing the effects of Ro 31-8220 and GF 109203X. The structure of GF 109203X is shown in (**A**) and Ro 31-8220 in (**B**). (**C**) Images of KBTeloMyoD fibroblast cells (DM1) are shown following treatment with Ro 31-8220 at 10 μm, DMSO and GF 109 203X at 40 μm. (**D**) The standard concentration curves for GF 109 203X treatment of KBTeloMyoD fibroblast cells in which the percentages of foci (green) and cell viability (blue) are represented relative to DMSO treatment. (**E**) Histogram showing nuclear and cytoplasmic distribution of MBNL1 following GF 109 203X treatment. The graph shows the combined data of triplicate samples for comparison of GF 109 203X and DMSO treatments. (**F**) The alternative splicing patterns of ATP2A1 obtained for untreated SBTeloMyoD differentiated fibroblasts (non-DM cells) (lane 1), KBTeloMyoD differentiated fibroblasts (DM1) treated with DMSO (lane 2) and KBTeloMyoD differentiated fibroblasts (DM1) treated with GF 109 203X (lane 3).

To establish if the effect of Ro 31-8220 treatment in our cell system is PKC dependent, we studied additional PKC inhibitor compounds. We tested a series of 12 structurally related compounds in the nuclear foci assay in both DM1 and DM2 fibroblast cells (Supplementary Material, Fig. S8A). At the very highest concentration (40 μm) two of the bisindolylmaleimides (VI and II) demonstrated a slight reduction in foci but had no effect at lower concentrations. All other PKC inhibitors tested had no effect across the 12 point concentration range (Supplementary Material, Fig. S8B and C). Interestingly, we observed that GF 109203X, although structurally very similar to Ro 31-8220, (Fig. 6A and B) demonstrated different effects on nuclear foci. Ro 31-8220 is effective at disrupting foci at a concentration of 2.5 μm, whereas GF 109203X has only a slight effect at the highest applied concentrations and no effect at concentrations <40 µm (Fig. 6C and D). The effect of GF 109203X was compared with that of Ro 31-8220 in two of the tertiary assays. We compared their effects on Muscleblind-like distribution and *ATP2A1* alternative splicing ratios. Ro 31-8220 changes the nuclear to cytoplasmic distribution of muscleblind, decreasing the amount of nuclear MBNL1 (Fig. 3C and E), whereas GF 109203X does not (Fig. 6E). Treatment with GF 109203X has no beneficial effect on the ratio of alternative splice isoforms of *ATP2A1* observed in the DM cell line KBTeloMyoD when compared with DMSO treatment (Fig. 6F). This contrasts with the effect of Ro 31-8220 which produces a correction in the splicing profile (Fig. 4). These data demonstrate that PKC inhibition by Ro 31-8220 is not responsible for the effects observed on MBNL-dependent events in our DM cell lines.

**Figure 7.**
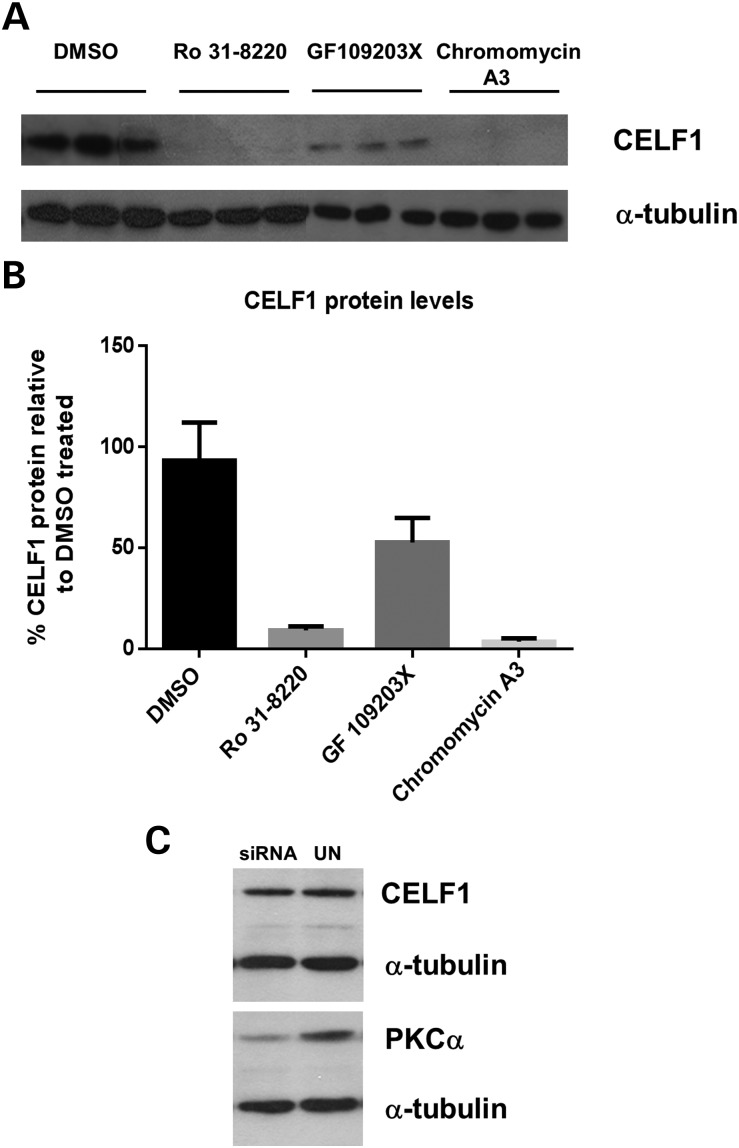
The effect of compound treatment on CELF1 protein levels. (**A**) CELF1 and α-tubulin protein levels were analysed on protein extracts from KB Telo MyoD cells (DM1) following treatment with DMSO, Ro 31-8220 (10 μm), GF 109203X (10 μm) and chromomycin A3 (40 nm). (**B**) Histogram shows quantification of CELF1 protein normalized against α-tubulin levels. (**C**) CELF1 protein levels were analysed in western blot analysis of untreated and PKCα knockdown extracts. PKCα inhibition was verified by western blot, normalized against α-tubulin.

Previous work has described the role of PKCα in a DM mouse model system and the destabilization of CELF1 protein following Ro 31-8220 treatment (36). To verify the effect of Ro 31-8220 and GF 109203X on CELF1 protein levels in our patient cell model, we conducted western blot analysis on triplicate samples following compound treatment. Consistent with previous reports treatment with Ro 31-8220 resulted in a 91% reduction in CELF1 protein. However, we found there to be only a 48% reduction in the level of this protein following treatment with GF 109203X (Fig. 7A and B). A comparison of the PKCα IC50 values for these two compounds demonstrates that GF 109203X is more potent on this target (IC50 8 nm) then Ro 31-8220 (IC50 33 nm) (37,38). To establish the effect of PKCα on CELF1 levels in our cell model, we performed siRNA knockdown of PKCα . We achieved 61% reduction of protein but found there to be no difference in CELF1 levels following knockdown, suggesting this kinase is not responsible for the results observed (Fig. 7C). Likewise, PKCα knockdown did not reduce the number of nuclear foci (data not shown). These data suggest that the effect on nuclear foci, MBNL-dependent events and CELF1 protein steady-state levels are not due to PKC inhibition in our cell system.

## DISCUSSION

The identification of compounds to treat inherited diseases represents a major challenge because the precise mechanism of pathophysiology for many of these rare conditions is still unclear and there has been a lack of major effort for drug development. Thus far, several approaches have been used to identify potential therapies for DM (23,24,26,27,38–40) including two cell-based studies using splice reporter systems (30,41). Although DM is often quoted to be a spliceopathy (42), in terms of the sequence of events underlying the disorder, splicing abnormalities arise at a later stage of the molecular pathway. One of the earliest events in DM pathophysiology occurs when repeat expansion RNA is transcribed and accumulates in the nuclei of DM cells, where it sequesters MBNL proteins, forming characteristic spots or foci (9). We have developed a phenotypic assay to screen for potentially useful therapeutic compounds for DM, based on the elimination of nuclear foci using an *in situ* hybridization protocol and high-content imaging of DM patient cell lines. We have screened four libraries including three that are commercially available and the NPC library. Although multiple hits were identified initially from the Chembridge Diverset™ library, none validated in the secondary screen, which combined a concentration/dilution series with tests for cell viability. In many cases the concentration range over which foci were affected, matched very closely the concentration range that killed the cells. Thus, the disappearance of foci in these cases was probably due to cell death. Surprisingly, pentamidine was not identified as a hit within the phosphatase inhibitor collection. This may be due to differences in cell lines and/or compound concentrations used in our experiments compared with those reported by Warf *et al*. (27) in which pentamidine was first identified as potentially useful for DM therapy.

Two compounds, chromomycin A3 from the NPC collection and Ro 31-8220 from the kinase inhibitor library, reduced foci in the primary screen and were validated in the secondary screen. The identification of chromomycin A3 is particularly interesting, as it has already been tested in clinical trials against various indications. Analysis of chromomycin A3 and Ro 31-8220 in further assays provided insights to their effect on other molecular features of DM. For example, treatment with the compounds produced a redistribution of MBNL1 protein, with a marked reduction in the nucleus, consistent with the observed reduction of MBNL protein in foci. Furthermore, both compounds produced a beneficial effect on the ratio of *INSR* and *ATP2A1* alternative splice isoforms, shifting the patterns observed in DM cells to be more like those observed in non-DM cells. However, although nuclear foci disappeared, as judged by *in situ* hybridization for RNA and immuno-cytochemistry for MBNL protein, the repeat expansion transcripts were not liberated to the cytoplasm and remain in the nucleus of DM cells. This indicates that the mutant transcripts are likely to be dispersed within the nucleus yet remain trapped, perhaps due to some intrinsic structural property of the repeat.

Nuclear foci consist of repeat expansion RNA complexed with MBNL and probably other proteins that have yet to be identified. Our assay identifies compounds that eliminate such foci. In effect, we are looking for a compound that will eliminate foci, restore the nucleo-cytoplasmic balance of MBNL proteins and correct DM-associated splicing abnormalities. Ideally, this would be accompanied by export of the repeat expansion transcripts or their degradation. However, it is not entirely clear whether all aspects of the molecular pathophysiology would have to be corrected for a compound to constitute a satisfactory therapy for DM. Both chromomycin A3 and Ro 31-8220 have some of the desired attributes of a DM therapy but further refinement of their activity is required. The underlying mechanism by which both compounds act on foci requires further investigation. Chromomycin A3 is a glycosidic antibiotic that inhibits the DNA-dependent RNA polymerase reaction by reversibly binding to G-C base pairs to inhibit RNA synthesis (43). As it is known that chromomycin, A3 interacts with GC-rich regions of duplex DNA (44,45), it is possible that it also interacts with stem-loop structures present in RNA foci. This binding interaction could result in overall disruption of the stem-loop structure, and hence facilitate the observed redistribution of MBNL1. One possible explanation for the effect of Ro 31-8220 treatment is that the nuclear/cytoplasmic localization of MBNL1 is determined by a phosphorylation event that is modified by Ro 31-8220. Additional work will be required to confirm this possibility.

In a zebrafish repeat expansion model, the proportion of phenotypically altered embryos was reduced following Ro 31-8220 treatment compared with non-treated embryos (Supplementary Material, Fig. S7). This is consistent with the beneficial effect of this inhibitor on the cardiac phenotype observed in a DM1 mouse model (37). Ro 31-8220 is a PKC inhibitor and recent work indicates that inappropriate activation of the PKC pathway is a factor in DM pathogenesis (13,37). Kuyumcu-Martinez *et al*. (13) have shown that PKC activation is detected 6 h after induction of repeat expansion transcripts in their mouse model and that PKC activation results in hyper-phosphorylation and increased steady-state levels of CELF1, although the phosphorylation site for PKC kinase within CELF1 remains to be determined. Furthermore, these authors show that DM mis-splicing events regulated by CELF1 are reduced by treatment with PKC inhibitor Ro 31-8220, whereas those regulated by MBNL1 are not. In the present study, however, we show that *ATP2A1* alternative splicing, which in muscle cells is MBNL1 dependent (35), is altered following treatment with Ro 31-8220. Thus, both CELF1- and MBNL1-related events are affected by treatment with Ro 31-8220. The sequestration of MBNL proteins in nuclear foci and CELF1 activation are key events in the molecular pathogenesis of DM, though the direct connection between the two has been difficult to establish. Our observations that Ro 31-8220 affects nuclear foci, MBNL1 distribution, *ATP2A1* splicing and CELF1 steady-state protein levels independently of PKC activity suggests an additional target of this kinase inhibitor that may link these two aspects of DM pathophysiology.

Kinase inhibitors are notoriously promiscuous and our study shows that Ro 31-8220 acts to affect nuclear foci, MBNL-dependent events and CELF1 protein levels independently of PKC, suggesting that another kinase is involved. Indeed work by others has shown the involvement of additional kinases in response to repeat expansion RNA (46–48). Our data demonstrate an unknown target of this compound and points to the need to identify the specific target for this activity. Understanding more about the activity of Ro 31-8220 may identify additional kinase involvement in the progression of the condition.

Thus, in summary, we report a medium throughput assay using *in situ* hybridization and high-content imaging to identify compounds that may provide the starting point for future drug development studies for DM therapy, and which provide possible alternatives to oligo-based approaches (25,28). The identification of Ro 31-8220 in our screen for compounds that affect nuclear foci demonstrates an important role for this compound on both MBNL- and CELF1-dependent events in DM, highlighting it as a good starting point for a drug discovery programme.

## METHODS AND MATERIALS

### Cell culture

Fibroblast cells were grown in Dulbecco's modified eagles medium (DMEM) with penicillin and streptomycin, and 10% foetal calf serum (FCS) (Sigma). To differentiate fibroblasts, the cells were plated in the same media with 1% FCS and 2 µg/ml doxycylin. Myoblast cultures were routinely grown in Ham's F10 containing penicillin and streptomycin with 20% FCS (GIBCO) and differentiated in DMEM containing 1% FCS. Repeat sizes were KBTeloMyoD—400 repeats, DM1400—1400 repeats, DM15—3000 repeats, DM16—800 repeats and KagoTelo—3000–5000 repeats.

### Small molecule libraries

Four different libraries were screened including 13 200 small molecules from the Chembridge Diverset™, 80 kinase inhibitors and 33 phosphatase inhibitors from Enzo Life Sciences. At the time of screening, the NCGC NPC consisted of 2724 small molecule compounds, 52% of which are drugs approved for human or animal use by the United States Food and Drug Administration, 22% are drugs approved in Europe, Canada or Japan, and the remaining 26% are compounds that have entered clinical trials or are research compounds commonly used in biomedical research (49). Information on all four libraries can be obtained at http://www.nottingham.ac.uk/~plzjdb/

### Compound exposure

Cells were aliquoted into 384-well plates with 1.6 × 10^4^ cells per well with each compound screened in triplicate. For the Chembridge Diverset library sequential dilution of compounds was performed on a Tecan Evo Liquid handling robot to produce a final concentration of 40 µm. For the NPC library, compounds were tested in single-pass at three concentrations; 40, 8 and 1.6 µm in 384-well format. The kinase and phosphatase libraries were screened in 96-well format with compound concentrations of 100, 10, 1 µm and 100 nm. Secondary validation screens of all libraries were performed over a 12-point dilution range, with a 1:3 dilution protocol, from 40 µm to 200 pm in 96-well format.

### *In situ* hybridization protocol

Cells were exposed to compounds for 48 h after which *in situ* hybridization was performed to identify foci using either a Cy3- or Cy5-labelled (CAG)_10_ probe for DM1 or (CAGG)_10_ probe for DM2. Plates were analysed on a Molecular Devices Micro High Content Imaging system, with nine fields imaged per well to give ∼100 cells per well, per compound treatment. The nuclear area was identified by Hoechst stain and the number, size and intensity of foci was determined by scoring adjacent pixels that were 80 greyscales or more above background. Data were exported to an excel spreadsheet and the means and standard deviations were calculated for the untreated controls for comparison with each treatment on the same plate. The *Z*-factor for this screening assay is 0.74 (*μ_n_* = 4.0682187, *μ_p_* = 0.06273117, σ*_n_* = 0.40122691 and *σ_p_* = 0.03129234).

### Assay for repeat expansion transcripts

Reverse transcription was performed using 1 μg total RNA from compound-treated and untreated cells. PCR was carried out using 1/20 of the synthesized cDNA with primers N11, 5′-CACTGTCGGACATTCGGGAAGGTGC and 133, 5′-GCTTGCACGTGTGGCTCAAGCAGCTG. For Genescan analysis primer N11 was labelled with FAM. Amplification was performed with a *T*_m_ of 58°C. The PCR product was subsequently heated to 95°C for 2 min followed by cooling to 4°C. For *Bpm*I restriction digestion analysis of DMPK PCR products, 4 μl of PCR mixture was digested overnight with restriction enzyme *Bpm*I (NEB) in a total reaction volume of 20 μl at 37°C. The final products were analysed by electrophoresis at 90 V with 3% agarose gels and the density of bands quantified using the ImageJ software or by fragment analysis on an ABI377 sequencer followed by Genescan quantification.

### Alternative splicing assays

One μg of total RNA, extracted from cultured cells, was used as a template for cDNA synthesis with random hexamers and M-MuLV reverse transcriptase (NEB). One μl of cDNA was used as a template for the PCR amplification with MegaMix (Microzone) and the following primers: ATP2A1 forward—5′-TTCGTTGCTCGGAACTACC and reverse—5′-GGTTGGGAAGGGGAATTTAC; INSR forward—5′-CCAAAGACAGACTCTCAGAT and reverse 5′-AACATCGCCAAGGGACCTGC. PCR amplification was performed under the following conditions: 95°C for 5 min, followed by 32 cycles of 95°C for 30 s, 60°C for 30 s, 72°C for 1 min and final extension at 72°C for 5 min; The PCR products were analysed by electrophoresis at 90 V with 3% agarose gels and the density of bands was quantified using the ImageJ software.

### Western blots and detection

Western blotting was performed using a commercial NuPage system (Invitrogen, UK) according to the manufacturer's instructions. The four primary antibodies used in this study were MB1a (50) (1:10 000 dilution), human CELF1 (3B1, Abcam, 1:2000 dilution), human α-tubulin and human Lamin B (both obtained from Santa Cruz and used at dilutions of 1:500). Anti-mouse IgG-horseradish peroxidase was used as the secondary antibody. The ImageJ software was used for the quantification of bands on western blots**.**

### Zebrafish model of myotonic dystrophy

The (CUG)_140_ repeat sequence was cloned into the pBluescript II SK plasmid and transcribed into RNA using the Ambion mMessage mMachine T7 Ultra kit. Embryos were injected with 50 pg (CUG)_140_ RNA. (CUG)_140_ repeat injected embryos and uninjected controls were treated with either 5 µm Ro 31-8220 or DMSO at 2 hpf. Compound was added directly to the fish water for 24 h. The resulting phenotype was monitored at 26 hpf. To ensure direct comparison measurements were established on somites from the yolk cell extension to the end of the embryo tail. All compound tests were conducted blind.

## SUPPLEMENTARY MATERIAL

Supplementary Material is available at *HMG* online.

## FUNDING

This research was funded by an MRC DPFS award, the Muscular Dystrophy Campaign, the Leverhulme Trust, the Myotonic Dystrophy Support Group, the Association Française contre les Myopathies, intramural research of the National Centre for Advancing Translational Sciences, NIH, and a Marigold Foundation Research fellowship for CZC. Funding to pay the Open Access publication charges for this article was provided by the Medical Research Council.

## Supplementary Material

Supplementary Data
