# Supplementary material for: High-content screening identifies small molecules that remove nuclear foci, affect MBNL distribution and CELF1 protein levels via a PKC-independent pathway in myotonic dystrophy cell lines
Source: Hum Mol Genet. 2013 Oct 30;23(6):1551–62. doi: 10.1093/hmg/ddt542 (PMC3929092; doi:10.1093/hmg/ddt542)
Supplement: Supplementary Data [file supp_23_6_1551__index.html]

High content screening identifies small molecules that remove nuclear foci, affect MBNL distribution and CELF1 protein levels via a PKC independent pathway in Myotonic Dystrophy cell lines — High-content screening identifies small molecules that remove nuclear foci, affect MBNL distribution and CELF1 protein levels via a PKC-independent pathway in myotonic dystrophy cell lines — High-content screening identifies small molecules that remove nuclear foci, affect MBNL distribution and CELF1 protein levels via a PKC-independent pathway in myotonic dystrophy cell lines — Supplementary Data 

# High-content screening identifies small molecules that remove nuclear foci, affect MBNL distribution and CELF1 protein levels via a PKC-independent pathway in myotonic dystrophy cell lines

## Supplementary Data

Supplementary Data

**Files in this Data Supplement:**

- Supplementary Data - Docx file
- Supplementary Figure 1 - TIF file
- Supplementary Figure 2 - TIF file
- Supplementary Figure 3 - TIF file
- Supplementary Figure 4 - TIF file
- Supplementary Figure 5 - TIF file
- Supplementary Figure 6 - TIF file
- Supplementary Figure 7 - TIF file
- Supplementary Figure 8A - TIF file
- Supplementary Figure 8B - TIF file
- Supplementary Figure 8Bi - TIF file
- Supplementary Figure 8C - TIF file
- Supplementary Figure 8Ci - TIF file
- Supplementary Table 1 - TIF file
- Supplementary Table 2 - TIF file
