# Supplementary material for: High-content screening identifies small molecules that remove nuclear foci, affect MBNL distribution and CELF1 protein levels via a PKC-independent pathway in myotonic dystrophy cell lines
Source: Hum Mol Genet. 2013 Oct 30;23(6):1551–62. doi: 10.1093/hmg/ddt542 (PMC3929092; doi:10.1093/hmg/ddt542)
Supplement: Supplementary Data [file supp_ddt542_ddt542supp.docx]

**Supplementary Methods**

**Cell Viability Assays**

Cell viability was assessed in the secondary screening assay using an MTT test. MTT (5mg/ml) was added to the cell culture media at 1/10^th^ the total volume. Cells were incubated for 3-4 hours at 37^o^C. The culture media was then removed and replaced with MTT solvent (0.1N HCl in anhydrous isopropanol) and mixed well to dissolve the MTT formazan. Absorbance readings were measured at 570nm subtracted from a background of 690nm on an Elisa plate reader. Prior to conducting the tertiary screens additional assays were conducted for cytotoxicity, apoptosis and a further cell viability screen in a Promega ApoTox-Glo Triplex Assay following the manufacturer’s protocol.

**Protein Extraction**

For total protein extraction, trypsinized cells were rinsed, pelleted, and stored at -80ºC prior to treatment with RIPA buffer for complete lysis. RIPA buffer consists of 50mM Tris, 150 mM NaCl, 0.1% SDS, 0.5% deoxycholic acid, 1% Triton X 100 or Nonident P40 and PMSF. For subcellular fractionation cells were scraped from the surface of a confluent 75cm^2^ flask in 500µl of ice cold PBS with 1mM PMSF and transferred to a 1.5ml eppendorf tube. The cell lysate was passed through a 25 gauge needle 10-12 times using a 1ml syringe and left on ice for 20 minutes. Nuclei were pelleted by centrifugation at 13,000 rpm for 10 minutes. The supernatant, the cytosolic fraction, was removed and stored at -20^0^C. The nuclear pellet was resuspended in 500µl of RIPA buffer and centrifuged at 13000 rpm for 2 minutes. The supernatant was transferred to a fresh eppendorf tube and stored as the nuclear fraction. Protein quantification was performed using either a Bradford assay or a Dc (Lowry) assay.

**siRNA transfection**

Cells were transfected with 2 siRNAs against PKCα, at a final concentration of 300nM, using the Lonza Basic Nucleofector kit for primary fibroblasts. Transfections were carried out using the Amaxa Nucleofector device. Cells were collected 3 days after transfection for total protein extraction.

**Supplementary Figure Legends**

**Supplementary Figure 1**

The strategy adopted to screen the Chembridge Diverset^TM^, the NCGC Pharmaceutical Collection and the kinase-inhibitor and phosphatase-inhibitor libraries involves four levels of assays.

**Supplementary Figure 2**

Outline of the overall approach to DM screening. Telomerized cells are treated with compounds in 384-well format and then subjected to *in situ* hybridization to visualize foci, which are scored on a Molecular Devices Micro high content imaging system. Images are analyzed using a customized journal and scored for number, size and intensity of nuclear foci.

**Supplementary Figure 3**

The effect of compound treatment on focus formation and survival over a range of concentrations from 40µM to 200pM are shown for four compounds that increase the number of foci. Gray line = DM1 Foci, Cyan line = DM2 Foci, Blue line = DM1 survival (ATP), Black = DM2 survival (ATP).

**Supplementary Figure 4**

The chemical structures of twelve nucleoside analogues that have no effect on focus formation.

**Supplementary Figure 5**

Histograms showing the effect of compound treatments on DM1 myoblasts, which produces a comparable reduction in numbers of foci to those observed in DM fibroblasts at equivalent concentrations.

**Supplementary Figure 6**

The effect of Ro 31-8220 and chromomycin A3 on protein foci is shown. Immunostaining with MBNL1a antibody reveals protein foci in DMSO treated KBTeloMyoD cells. Protein foci are not detected in non-DM cells. Cells treated with Ro 31-8220 and chromomycin A3 either lack, or have very much reduced numbers of foci as shown in the histogram.

**Supplementary Figure 7**

**Partial rescue of the CUG_140_ phenotype following treatment of Zebrafish embryos with RO-31-8220.** Wild type **(A,B)** and CUG_140_ injected **(C,D)** embryos are shown. Phenotypic assessment of treated embryos was based on the number of somites beyond the yolk cell extension **(E)** represented as a percentage relative to uninjected controls. **(F)** The length to width ratio of the embryo tail is also shown as a percentage relative to uninjected controls. Measurements were conducted at 26hpf and 24 hours post compound treatment.

**Supplementary Figure 8**

**(A)** The chemical structures are shown for twelve PKC inhibitors tested for their effects on nuclear foci. **(B,C)** show the standard concentration curves for twelve different Bisindolylmaleimides following treatment of KBTeloMyoD cells (DM1) **(B)** and KAGOTelo cells (DM2) **(C)** in which the percentages of foci (green) and cell viability (blue) are represented relative to DMSO treatment on the ‘y’ axis and compound concentration (log μM) is indicated on the ‘x’ axis.
