# Supplementary figures and images for: High-content screening identifies small molecules that remove nuclear foci, affect MBNL distribution and CELF1 protein levels via a PKC-independent pathway in myotonic dystrophy cell lines
Source: Hum Mol Genet. 2013 Oct 30;23(6):1551–62. doi: 10.1093/hmg/ddt542 (PMC3929092; doi:10.1093/hmg/ddt542)

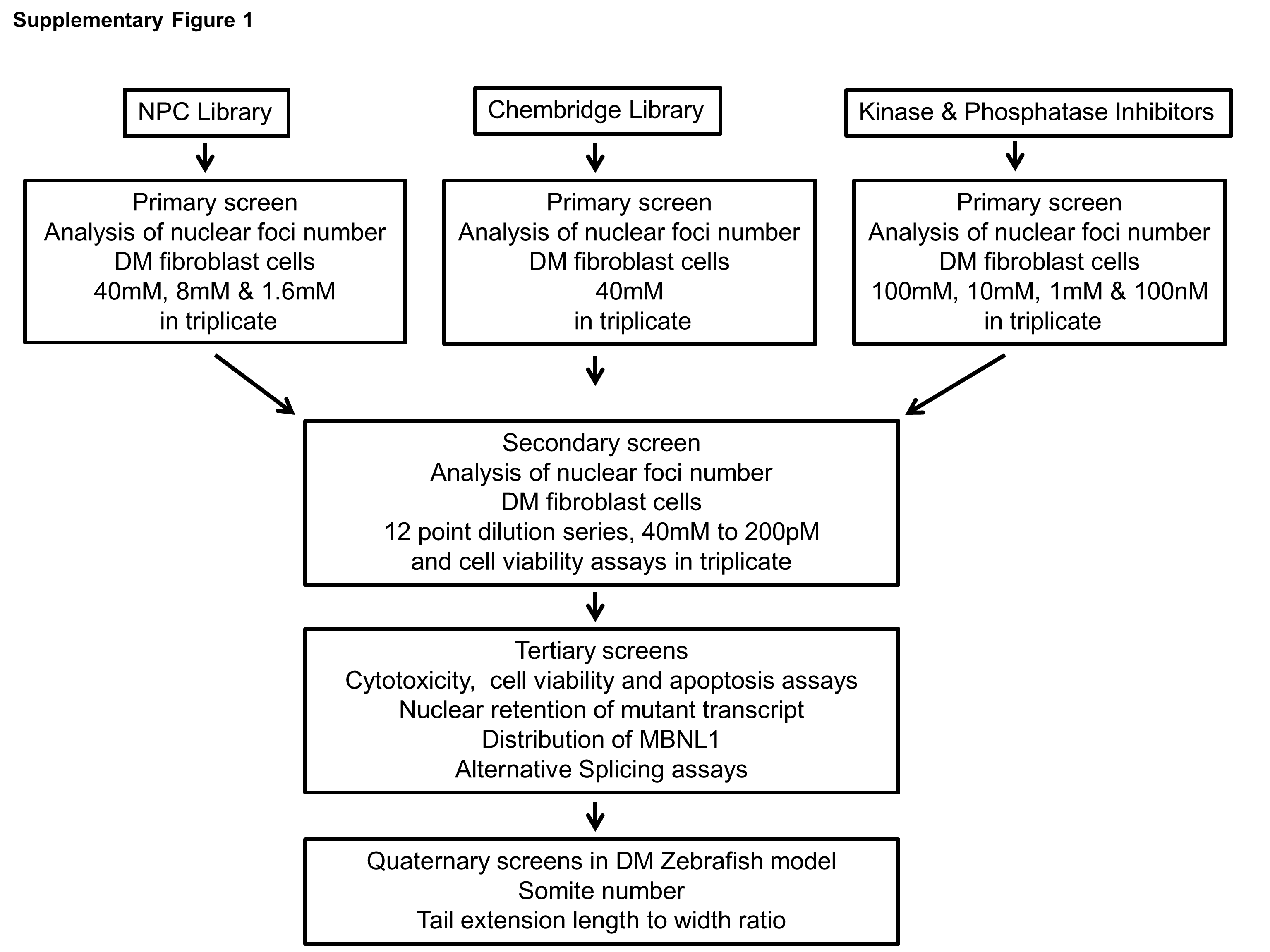

Supplement: Supplementary Data [file supp_ddt542_ddt542supp_fig1.tif]

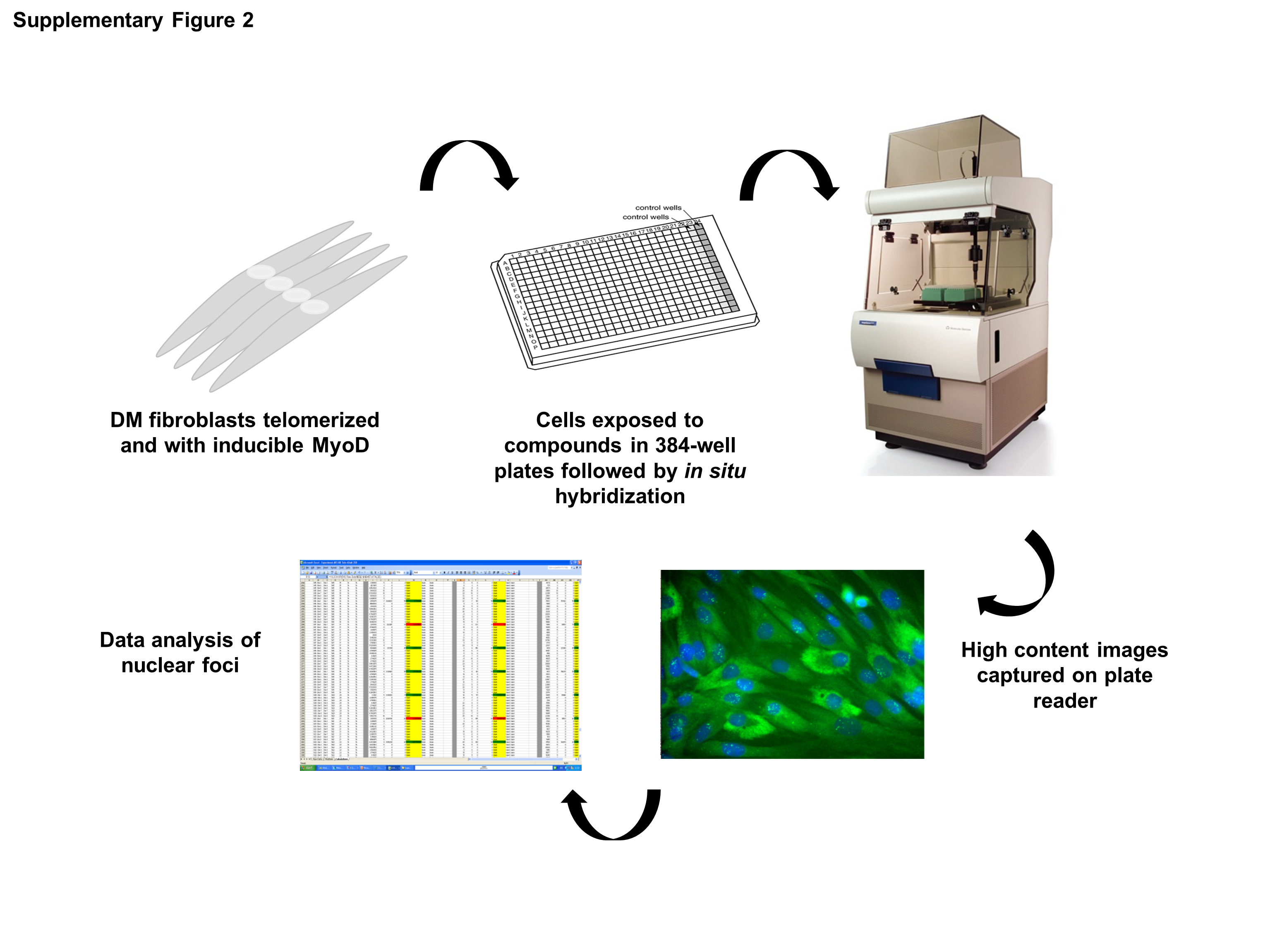

Supplement: Supplementary Data [file supp_ddt542_ddt542supp_fig2.tif]

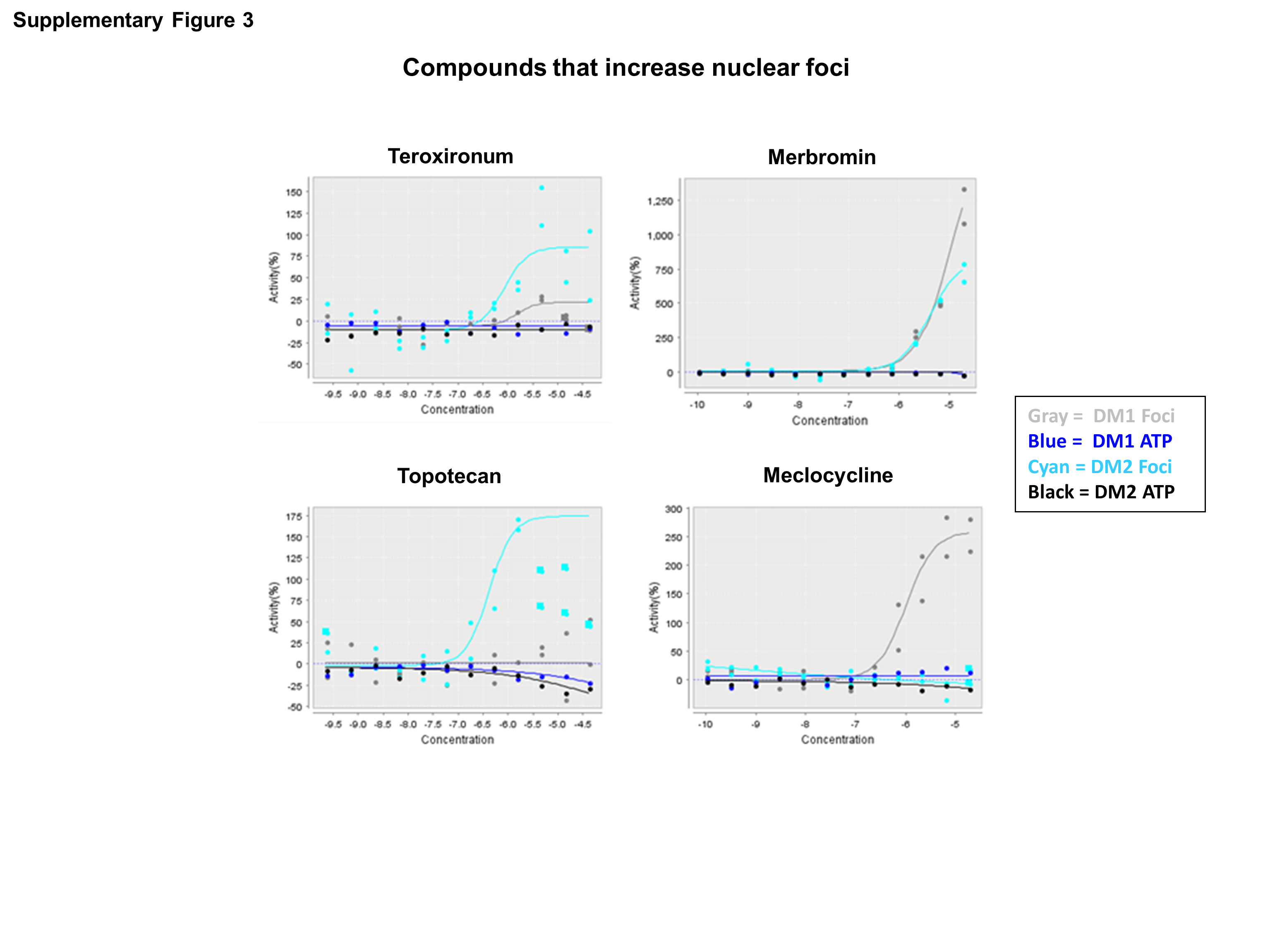

Supplement: Supplementary Data [file supp_ddt542_ddt542supp_fig3.tif]

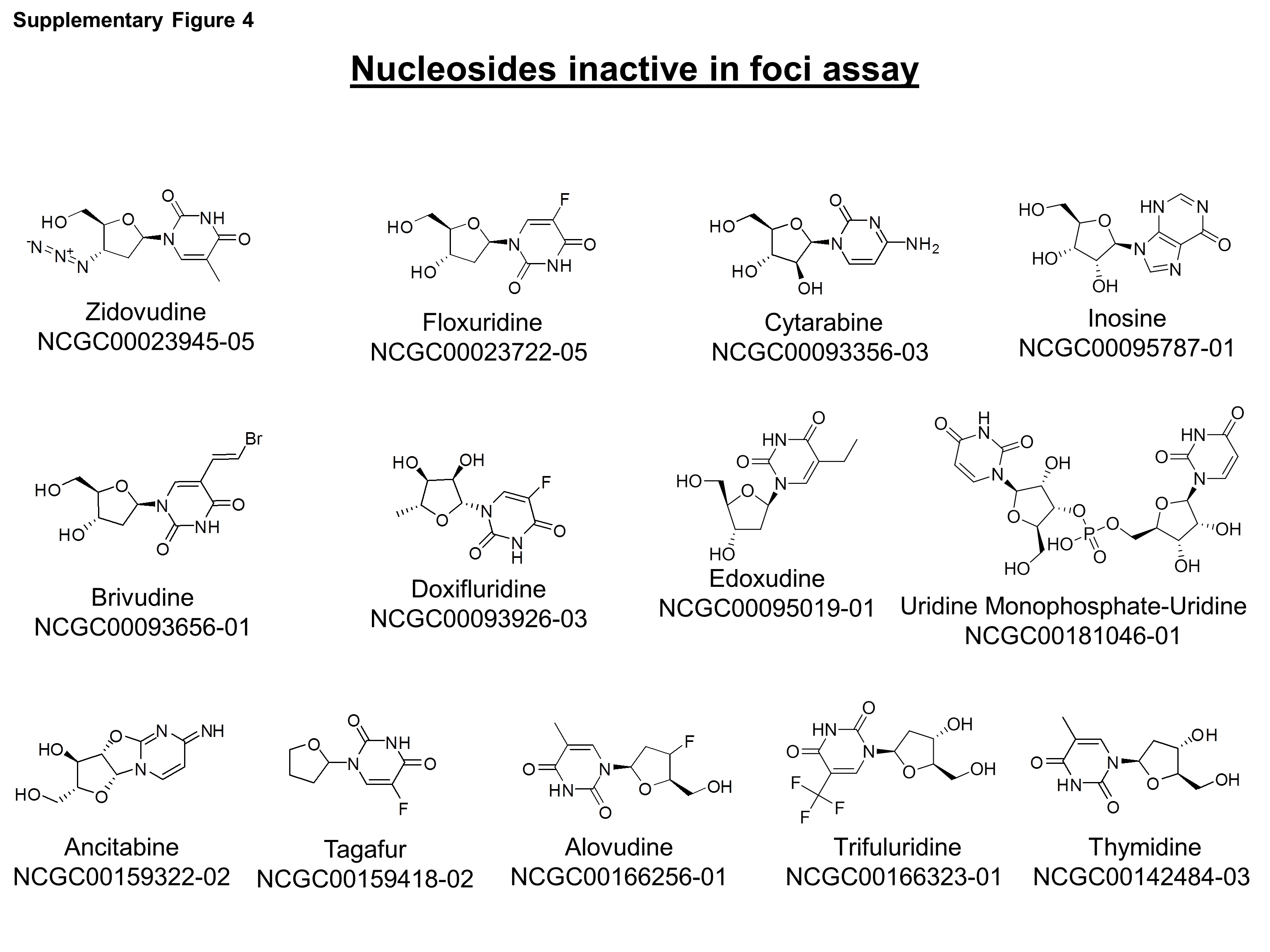

Supplement: Supplementary Data [file supp_ddt542_ddt542supp_fig4.tif]

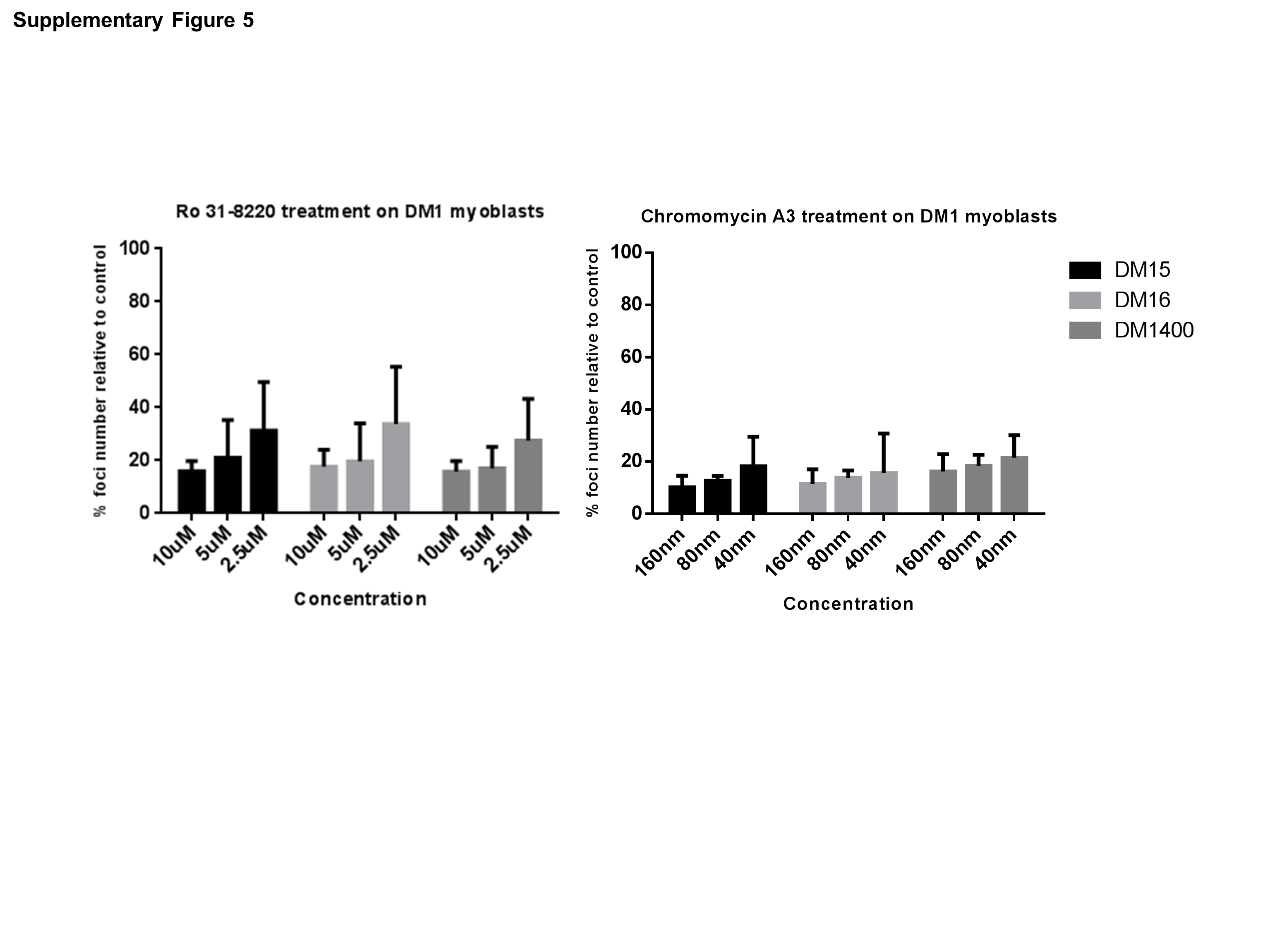

Supplement: Supplementary Data [file supp_ddt542_ddt542supp_fig5.tif]

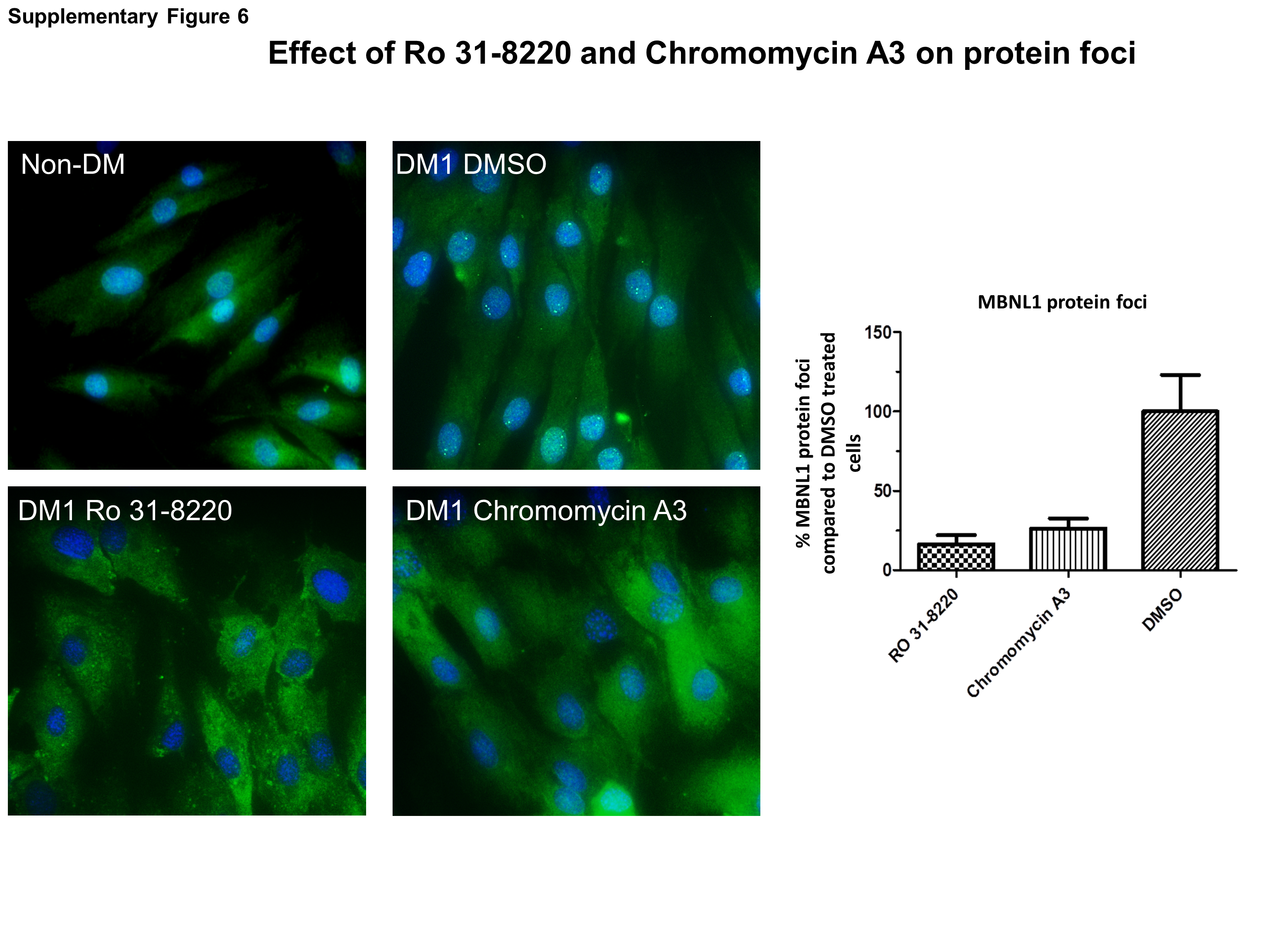

Supplement: Supplementary Data [file supp_ddt542_ddt542supp_fig6.tif]

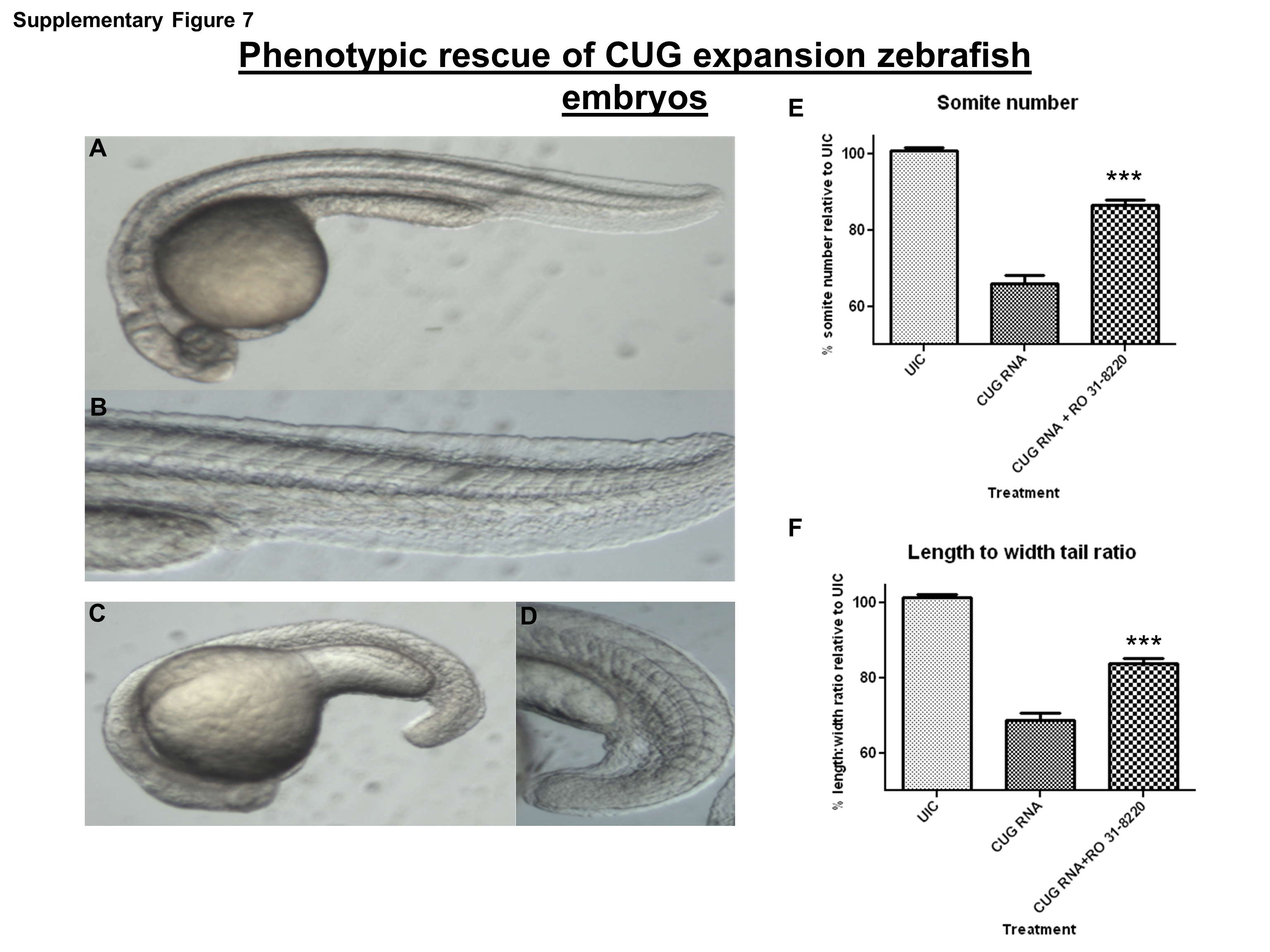

Supplement: Supplementary Data [file supp_ddt542_ddt542supp_fig7.tif]

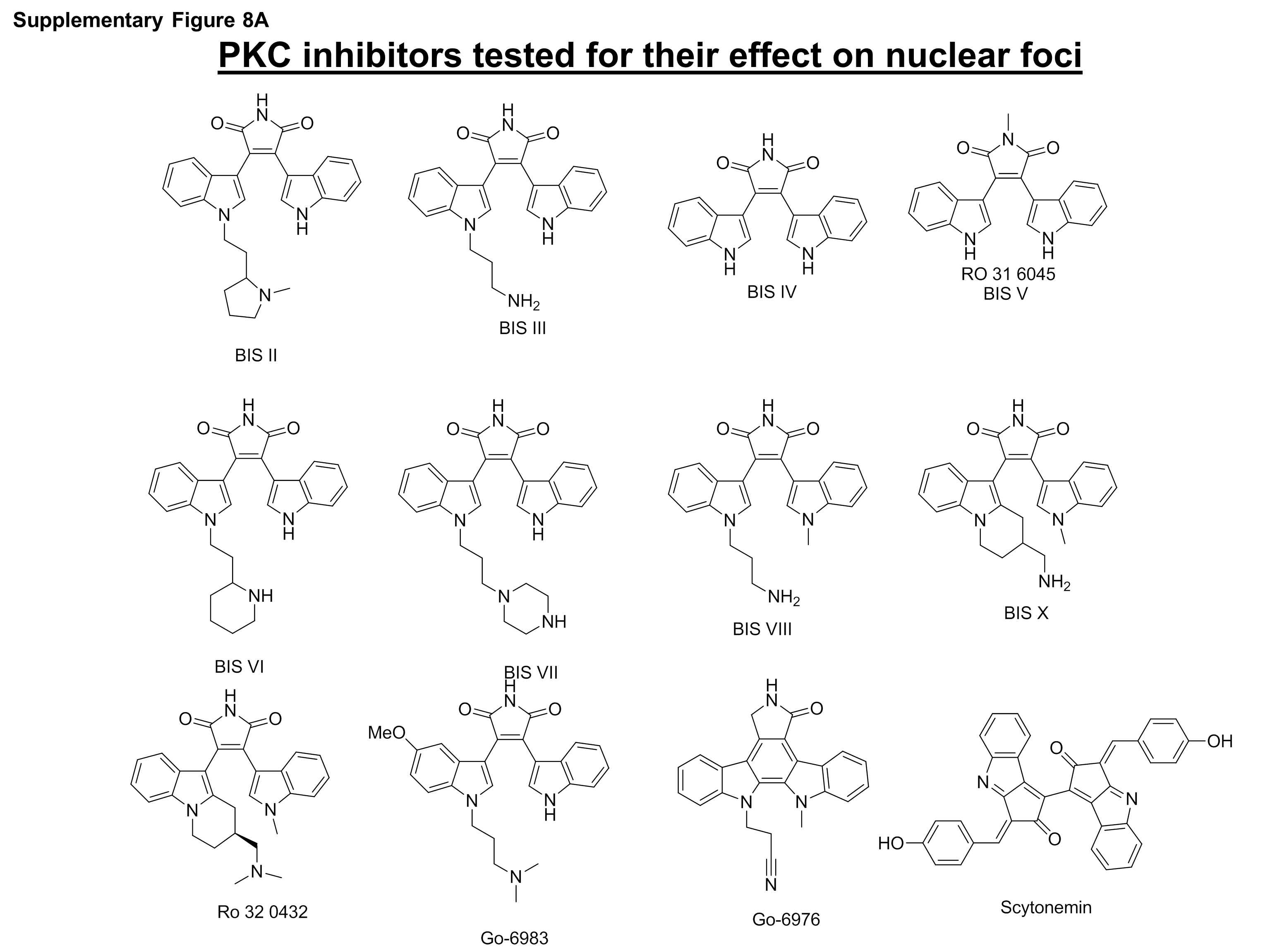

Supplement: Supplementary Data [file supp_ddt542_ddt542supp_fig8A.tif]

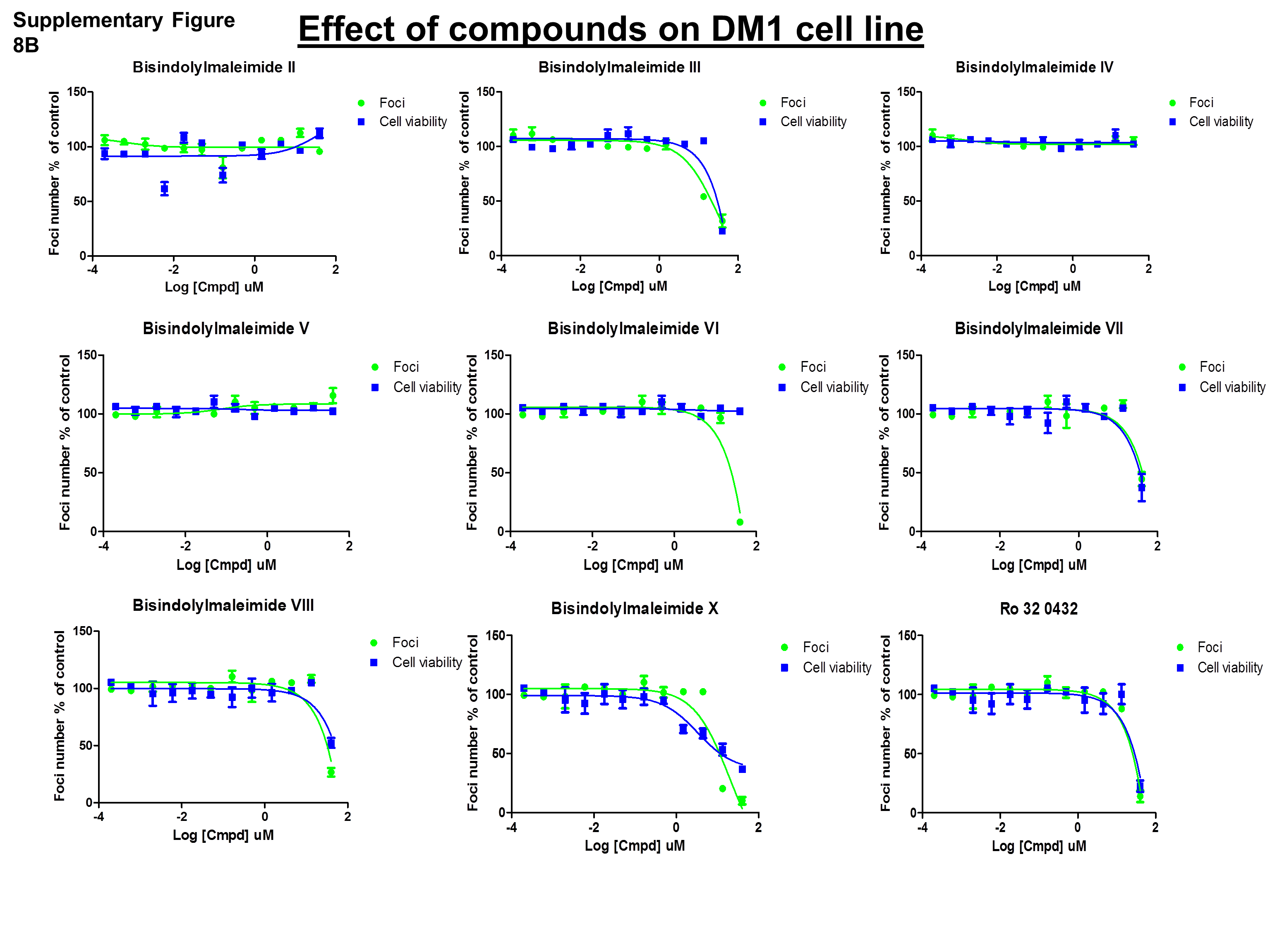

Supplement: Supplementary Data [file supp_ddt542_ddt542supp_fig8B.tif]

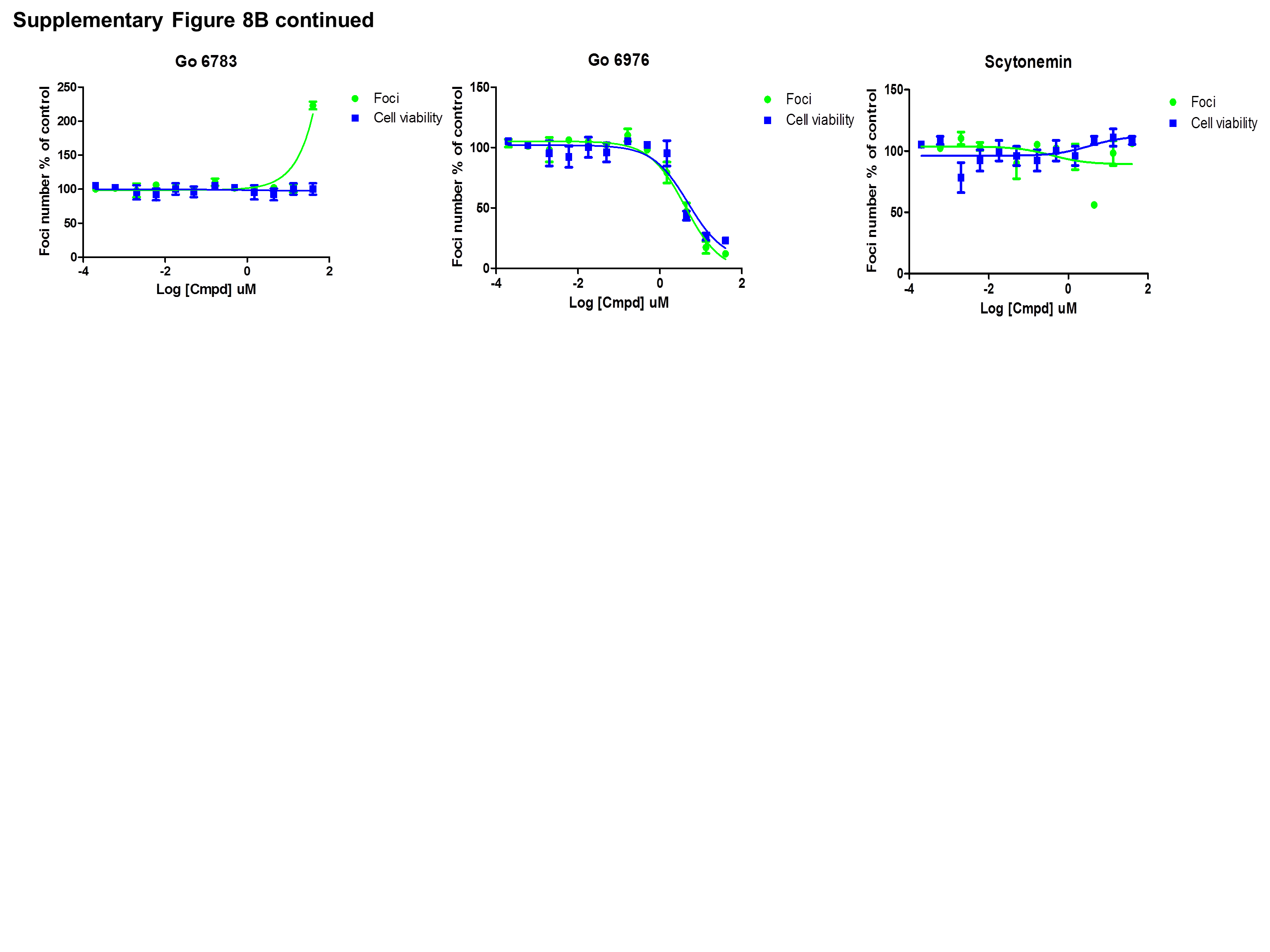

Supplement: Supplementary Data [file supp_ddt542_ddt542supp_fig8Bi.tif]

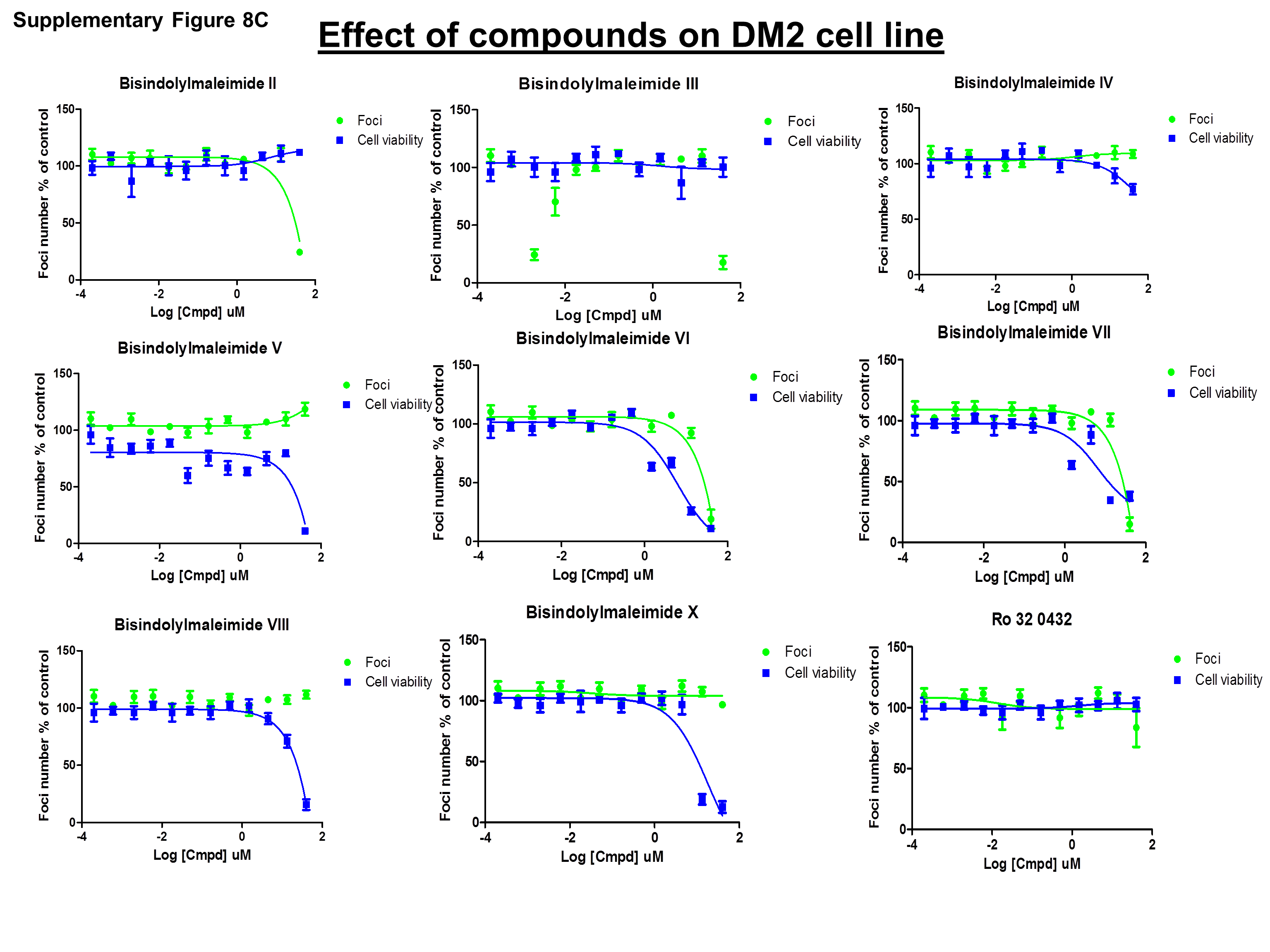

Supplement: Supplementary Data [file supp_ddt542_ddt542supp_fig8C.tif]

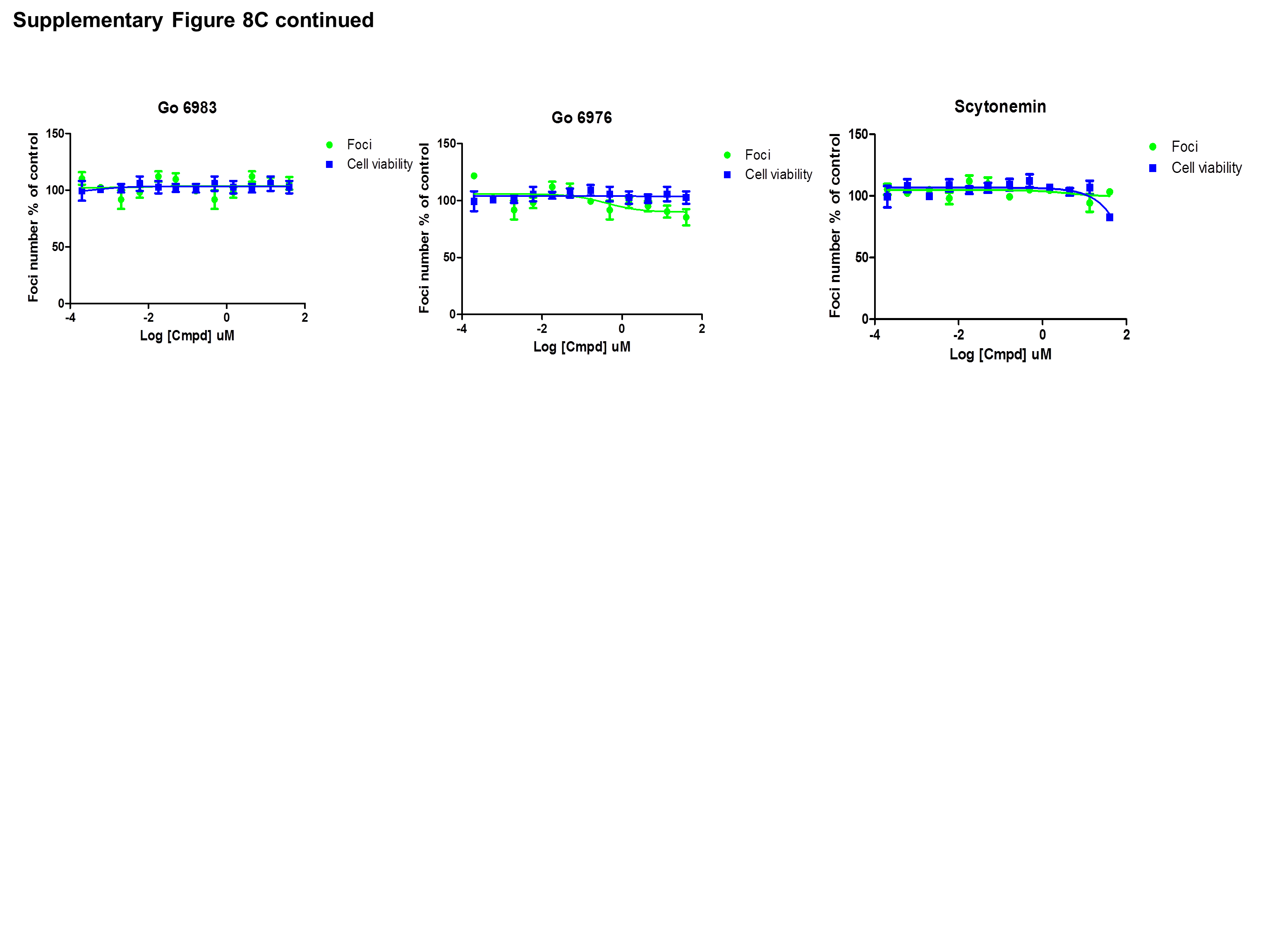

Supplement: Supplementary Data [file supp_ddt542_ddt542supp_fig8Ci.tif]

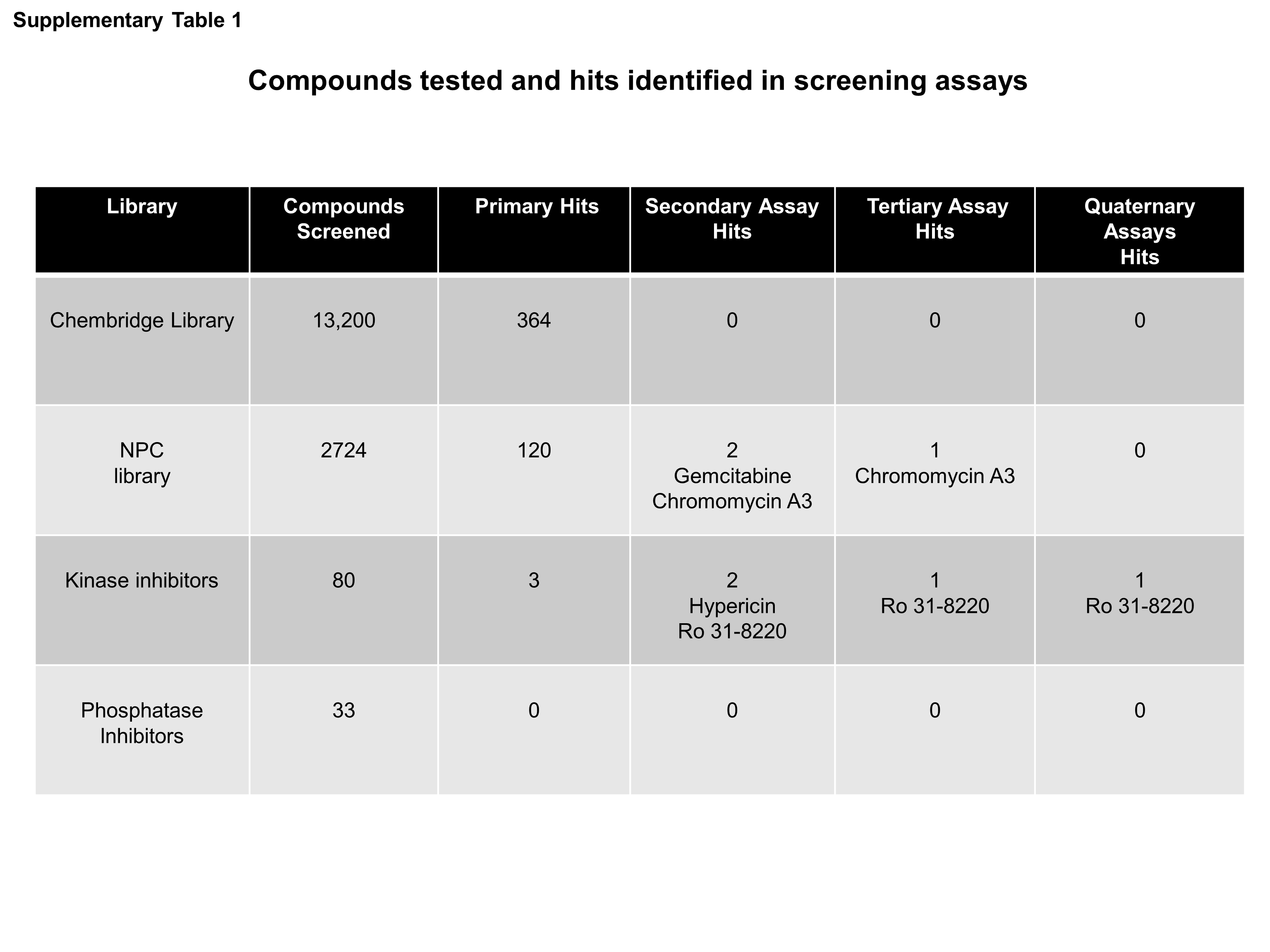

Supplement: Supplementary Data [file supp_ddt542_ddt542supp_table1.tif]

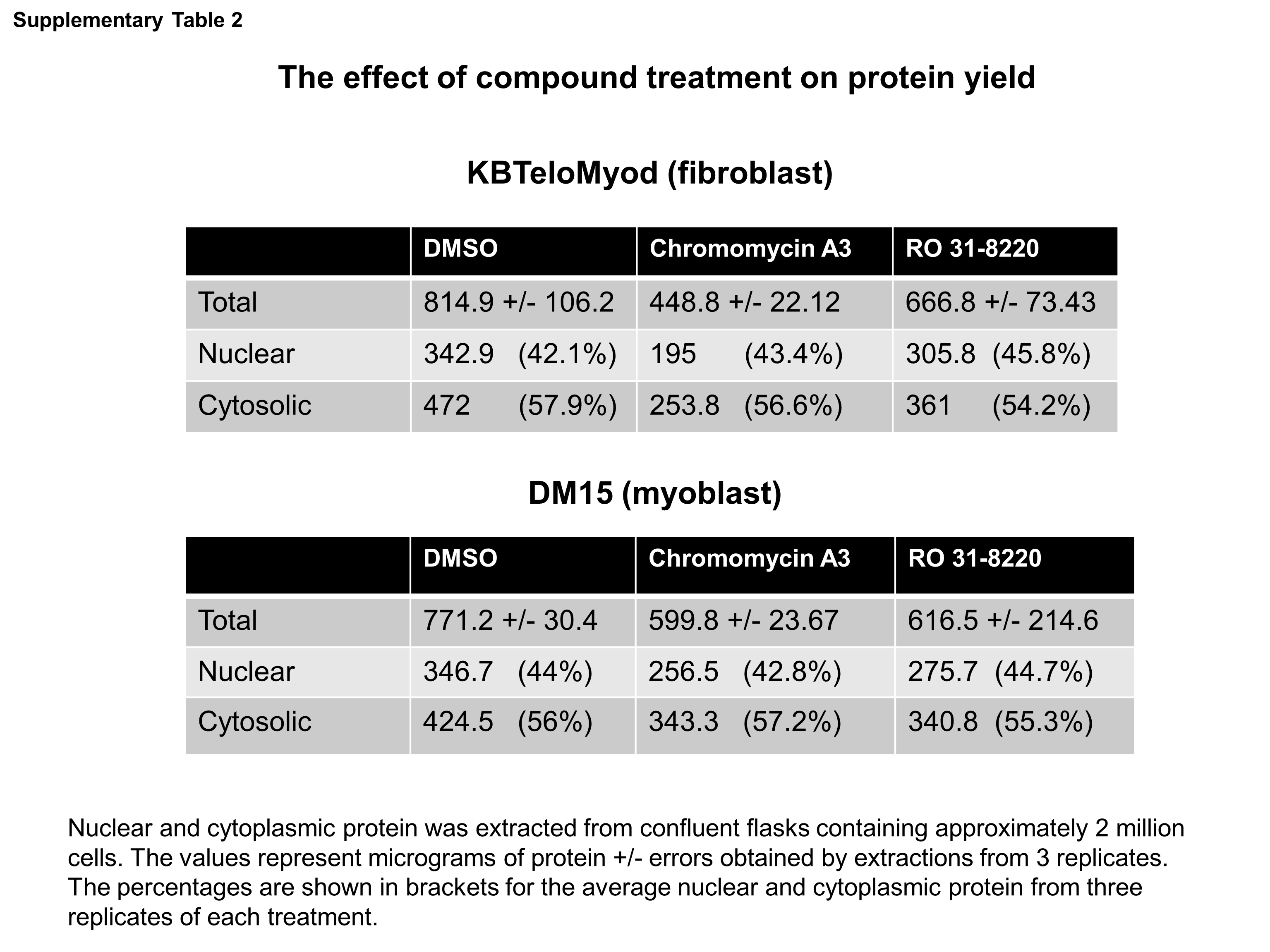

Supplement: Supplementary Data [file supp_ddt542_ddt542supp_table2.tif]
